# Proteomic analysis of the carotenogenic yeast *Xanthophyllomyces dendrorhous*

**DOI:** 10.1186/1471-2180-11-131

**Published:** 2011-06-13

**Authors:** Pilar Martinez-Moya, Steven Alexander Watt, Karsten Niehaus, Jennifer Alcaíno, Marcelo Baeza, Víctor Cifuentes

**Affiliations:** 1 Departamento de Ciencias Ecológicas, Centro de Biotecnologia, Facultad de Ciencias, Universidad de Chile, Santiago, Chile; 2 Department of Proteome and Metabolome Research, Faculty of Biology, Bielefeld University, Bielefeld, Germany

## Abstract

**Background:**

The yeast *Xanthophyllomyces dendrorhous *is used for the microbiological production of the antioxidant carotenoid astaxanthin. In this study, we established an optimal protocol for protein extraction and performed the first proteomic analysis of the strain ATCC 24230. Protein profiles before and during the induction of carotenogenesis were determined by two-dimensional polyacrylamide gel electrophoresis and proteins were identified by mass spectrometry.

**Results:**

Among the approximately 600 observed protein spots, 131 non-redundant proteins were identified. Proteomic analyses allowed us to identify 50 differentially expressed proteins that fall into several classes with distinct expression patterns. These analyses demonstrated that enzymes related to acetyl-CoA synthesis were more abundant prior to carotenogenesis. Later, redox- and stress-related proteins were up-regulated during the induction of carotenogenesis. For the carotenoid biosynthetic enzymes mevalonate kinase and phytoene/squalene synthase, we observed higher abundance during induction and/or accumulation of carotenoids. In addition, classical antioxidant enzymes, such as catalase, glutathione peroxidase and the cytosolic superoxide dismutases, were not identified.

**Conclusions:**

Our results provide an overview of potentially important carotenogenesis-related proteins, among which are proteins involved in carbohydrate and lipid biosynthetic pathways as well as several redox- and stress-related proteins. In addition, these results might indicate that *X. dendrorhous *accumulates astaxanthin under aerobic conditions to scavenge the reactive oxygen species (ROS) generated during metabolism.

## Background

The basidiomycete *Xanthophyllomyces dendrorhous *(formerly known as *Phaffia rhodozyma*) is an excellent astaxanthin-producing yeast and has been regarded as one of the most promising microorganisms for the commercial production of this carotenoid [1,2]. Astaxanthin is a pigment that produces the characteristic coloration of some birds, crustaceans and salmon. It has been used as a feed and food pigment in the aquaculture industry and has been evaluated as a pharmaceutical component because it may possess antioxidant activity [3,4]. Due to its biotechnological significance, investigations have been performed to improve astaxanthin production by optimizing fermentation methodologies [5,6] selecting for over-producing strains [7,8], using chemical stimulants [9,10], and employing genetic and metabolic engineering [11-13].

In *X. dendrorhous*, astaxanthin is produced via the mevalonate pathway, in which acetyl-CoA is a precursor to the formation of isopentenyl pyrophosphate (IPP), the general precursor of all isoprenoids. Chain elongation by successive head-to-tail condensation is then catalyzed by prenyltransferases with different chain length specificities [14] to generate carotenoid precursors. Thus, farnesyl pyrophosphate (FPP) (C15) and geranyl pyrophosphate (GGPP) (C20) are the immediate precursors of C30 and C40 carotenoids. GGPP formation is catalyzed by a GGPP synthase. The condensation of two molecules of GGPP is catalyzed by the bifunctional enzyme phytoene synthase to produce phytoene (C40). Lycopene is generated by phytoene desaturase, which introduces four double bonds into phytoene. A bifunctional enzyme with a lycopene cyclase activity then transforms lycopene into β-carotene by two cyclization reactions. Finally, β-carotene is oxidized by astaxanthin synthetase to yield astaxanthin [15].

Because little information on the genomics and regulation of carotenogenesis in *X. dendrorhous *is available, studies of astaxanthin production from a genetic perspective have been hampered; however, an alternative approach to address these biological questions is proteomics Two-dimensional (2D) techniques are the most generally applicable methods for obtaining a global picture of protein expression levels, and mass spectrometry (MS) has become the technology of choice for protein identification [16,17]. In previous studies, it has been demonstrated that 2D electrophoresis coupled with peptide mass fingerprinting (PMF) is a viable approach for the identification of homologous proteins across species boundaries [18-21]. Therefore, biosynthetic pathways and metabolic events in *X. dendrorhous *may be deduced from the functions of previously identified proteins.

In the present study, we used 2D protein electrophoresis coupled with matrix-assisted-laser-desorption/ionization time-of-flight mass spectrometry (MALDI-TOF MS) to analyze soluble protein extracts from *X. dendrorhous *cells grown on glucose minimal medium (MM-glucose). To the best of our knowledge, this is the first proteomic study on this yeast; thus, prior to protein characterization, we designed an optimized protocol for protein extraction. Because some specific or late reactions in carotenogenesis involve membrane-bound enzymes, we designed a protocol for the enrichment of membrane-bound proteins. These extracts were separated in two dimensions to obtain a protein map. In our analysis, the most abundant proteins were involved in primary metabolic pathways, and carbohydrate and lipid metabolic proteins showed the highest intensity spots. Interestingly, along with some carotenogenesis proteins, redox- and stress-associated proteins were up-regulated. This proteomic study is an important starting point and may be a useful reference for further studies of metabolic pathways, especially astaxanthin synthesis in *X. dendrorhous*.

## Results and discussion

### Isolation of soluble proteins and 2D electrophoresis

The aim of the present study was to characterize the proteome of soluble protein extracts of the yeast *X. dendrorhous *grown in MM-glucose media. Therefore, sample preparation prior to the IEF run was important for optimal electrophoretic separation of proteins.

In the Methods, we describe the comprehensive protocol used to obtain the soluble protein extracts. Briefly, to improve cell disruption and minimize proteolysis, lyophilized yeast cells were vortexed directly with glass beads. Lysis buffer and protease inhibitors were then added to reduce proteolytic enzyme activity. The pellet was disrupted five times in a RiboLyzer, followed by phenol extraction and methanol precipitation. Finally, the protein spots were stained with Coomassie and identified by MALDI-TOF MS.

**Figure 1:**
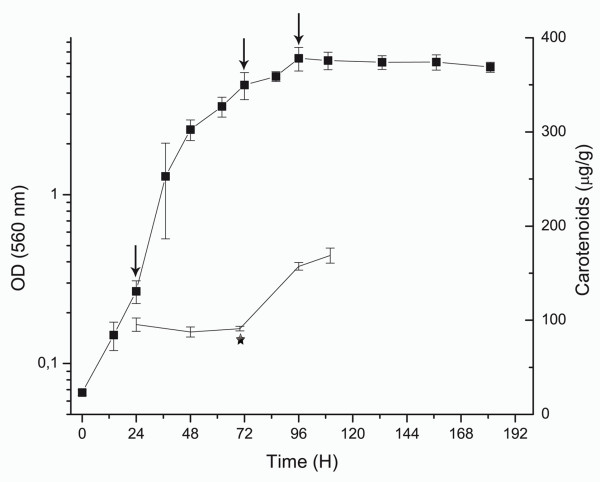
**Growth and pigment production in *X. dendrorhous***. Growth was measured by the absorbance at 560 nm (shown on a log scale), which is represented by the squares and solid line. The means ± SD of the values obtained from four independent cultures are shown. The vertical arrows indicate the harvest times for the assays (24, 70 and 96 h, which corresponded to lag, late exponential and stationary growth phases, respectively). The solid line represents the total carotenoids. The asterisk indicates the induction of carotenoid biosynthesis.

To obtain the protein profiles of *X. dendrorhous*, the yeast was cultured in MM-glucose and harvested at the lag, late exponential and stationary growth phases. Four independent cultures showed continuous increases in cell density until 70 h, which was immediately prior to the induction of carotenoid biosynthesis (Figure 1). As we have previously reported, pigment accumulation in MM-glucose was evident during the stationary phase [22,23]. Carotenoid analysis by HPLC showed that astaxanthin was the main carotenoid (75-90% of the total carotenoids) produced by the yeast during growth.

For the proteomic analyses, triplicate protein extracts (prepared from three independent cultures) were subjected to 2D analysis, and their protein profiles were obtained. The different protein profiles were subjected to a stringent comparative analysis using PDQuest software (version 7.1.1, Bio-Rad). After automated spot detection, spots were checked manually to eliminate possible artifacts such as background noise or streaks. Student's t-test (p < 0.05) was used to determine whether the relative changes in protein abundance were statistically significant.

**Figure 2:**
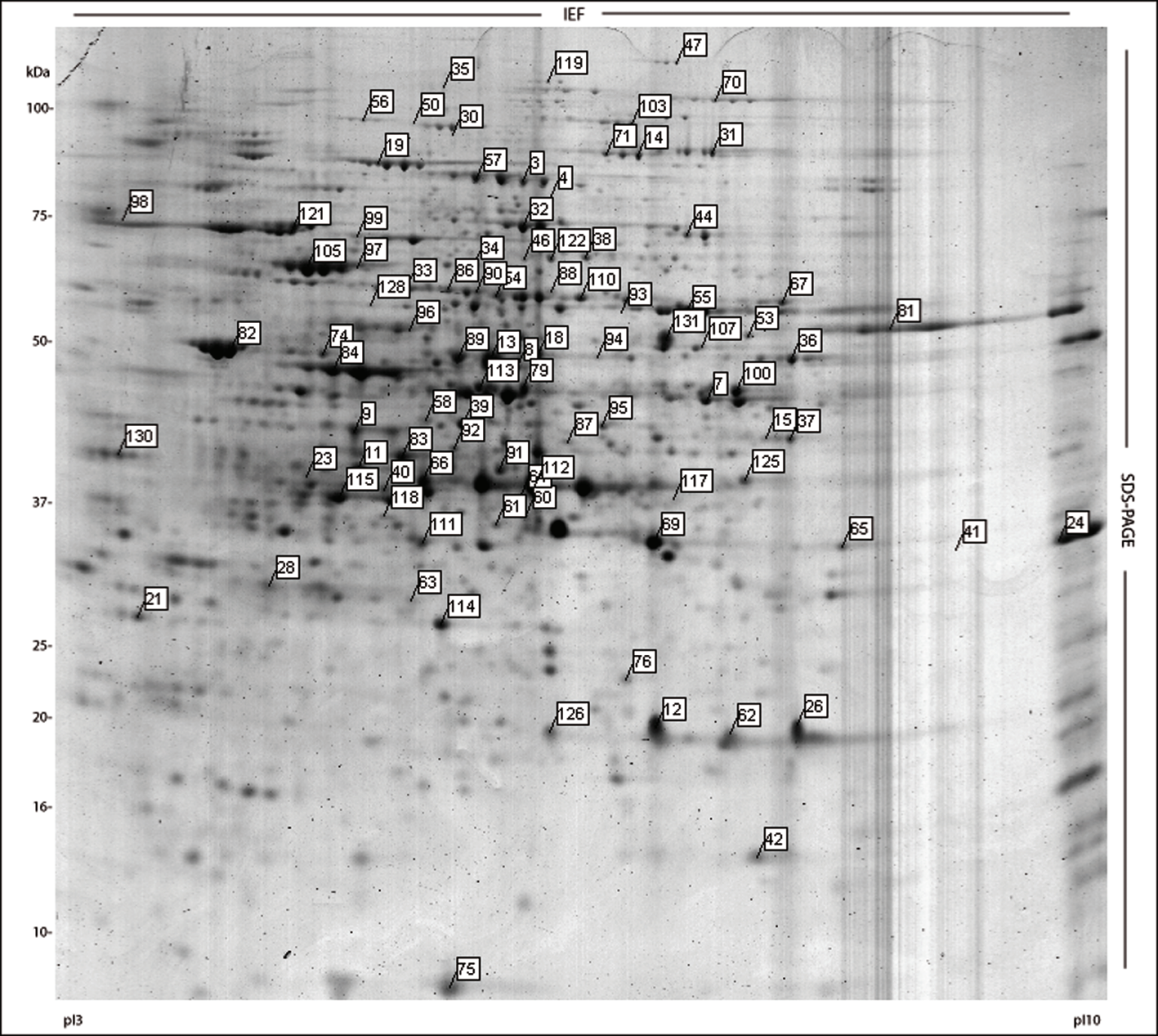
**Representative 2D gel of soluble proteins of *X. dendrorhous***. Protein profile in stationary growth phase. The image was obtained with PDQuest software ver. 7.1.1. The ID numbers were manually added and correspond to all non-redundant proteins identified by MALDI-TOF MS.

A representative 2D image is shown in Figure 2. The protein data analyses showed a consistent protein profile during growth (See additional file 1, Fig. S1). On average, approximately 600 spots were detected on each 2D gel in a pI-range of 3-10 and a molecular mass range of 10-100 kDa. This pattern of proteins was highly reproducible, and similar results were obtained in the triplicate cell extracts. Overall, the protein profiles did not change dramatically (over 90% of the spots were identical) during growth. Of the spots detected in all gels, 450 spots with different intensities were selected to be excised, digested with trypsin and analyzed by MALDI-TOF MS for protein identification. In total, 131 non-redundant proteins (we only considered one protein that was present in multiple spots) were identified in 171 spots (see additional file 2, Table S1). The proteins identified were classified according to their biological functions. Because it was impossible to determine the spot intensities for overlapping spots, we only quantified 161 single-protein spots.

### Evaluation of multiple spots and differentially migrating proteins

Proteins expressed from a single gene can migrate to multiple spots on 2D gels due to either post-translational modifications (such as chemical modification, proteolytic processing, and covalent attachment of small adducts) or artifactual modifications. It has been reported that several yeast proteins are resolved in multiple spots on 2D gels [24,25]. Consistent with these findings, we identified 22 proteins that were represented by multiple spots (see additional file 2, Table S1), and approximately 10 proteins were present in more than three spots (Figure 2 and additional file 1, Fig. S1), including the stress-related proteins HSP70 (protein N°99) and ATP synthase β (protein N°82), which were previously reported to have multiple spots [26,27], and PP1-1 (protein N°19), a protein that regulates the cellular response in glucose starvation and stress [28].

In most cases, multi-spot proteins showed charge variations (horizontal spot patterns, Figure 2 and additional file 1, Fig. S1), which are usually due to protein phosphorylation or other post translational modifications that alter the isoelectric point of a protein [29]. Interestingly, we found the protein, methionyl-tRNA formyltransferase (protein N°69), that had a diagonal spot pattern, which is less frequently reported (Figure 2 and additional file 1, Fig. S1). This migration pattern agrees with the results of previous studies [24-27,30], in which several metabolic proteins displayed distinct migration patterns. These multiple electrophoretic species could be generated by proteolytic events or could represent isoenzymes [29]; these possibilities were not further investigated in this work.

**Figure 3:**
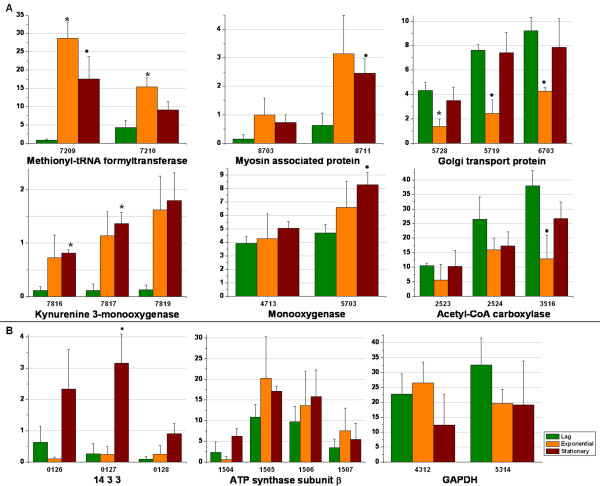
**Relative intensities of multiple spots for *X. dendrorhous *proteins in MM-glucose**. The growth phases are represented by different colors. **A**. Multi-spot proteins that exhibited essentially the same general pattern of variation. **B**. Multi-spot proteins that were regulated in different ways. The axis numbers correspond to the SSP spot identifications generated by PDQuest software. The y axis scale (× 10^3^) corresponds to the normalized spot intensity. To normalize, the spot intensities were divided by the total density of valid spots and then multiplied by 10^6^. Finally, the normalized values from replicates of 24-h, 70-h and 96-h were averaged. Asterisks represent p < 0.01 and circles represent p < 0.05.

Approximately 25% of the proteins identified in this study had potential posttranslational modifications or belonged to multigenic protein families. Accordingly, we studied the intensity profiles of proteins with multiple spots (Figure 3), of 22 multi-spot proteins identified 8 subgroups of proteins share similar profiles. For instance, a higher abundance of methionyl-tRNA formyltransferase and myosin-associated protein were observed at the end of the exponential phase. (Figure 3A). In contrast, acetyl-CoA carboxylase and Golgi transport protein showed dramatic decreases in spot intensity in the late exponential phase. Monooxygenase and kynurenine 3-monooxygenase showed increasing intensities during growth. Moreover, other sets of spots that corresponded to the same protein were notably different (Figure 3B), suggesting that the isoforms are regulated in different ways or are involved in different physiological processes. This form of regulation has been previously reported for some proteins involved in carbohydrate metabolism [16,31]. Unfortunately, no data could be extracted from our MALDI-TOF analyses to identify differences between the probable isoforms identified.

Regarding the migration of proteins, for which full *X. dendrorhous *sequences were available, the experimental Mr and pI values corresponded closely to the theoretical values, except for acetyl-CoA carboxylase (N°84). For this protein, the experimental Mr was markedly lower than the theoretical Mr. This discrepancy in Mr could be linked to either in vivo or in vitro protein degradation. In fact, this protein was identified with peptides that spanned the middle and carboxy terminal regions of the reported amino acid sequence. However, for the orthologous proteins identified, we found reasonable correlations between the experimental and theoretical migrations (see additional file 2 Table S1). Most discrepancies corresponded to a lower Mr value and more acidic pI value for the gel-estimated value compared to the theoretical value. For instance, phosphatidylserine decarboxylase (protein N°85) was detected in the acidic range (pI 6.24), but this protein has a basic theoretical pI of 9.45. This unusual migration has been observed in ribosomal proteins in previous studies [30]; while this behavior still has no explanation, it is probably related to the presence of posttranslational modifications.

### Protein identification and classification into functional groups

We employed the approach of cross-species protein identification for *X. dendrorhous *because this yeast is poorly characterized at the gene and protein levels. The conserved nature of many biosynthetic and metabolic pathways in different organisms has been the basis for several studies of species that lack genome sequence data [18,20,21]. Furthermore, based on theoretical and experimental evidence, it has been shown that sequence identities greater than 60% are sufficient to match proteins with high confidence across species boundaries [20,21]. It fact, it has been previously reported that conserved structural motifs could be identified across distant species with total amino acid sequence identities as low as 29.6% [18]. In this work, nitrite reductase was identified with 14 mass peptides that covered 16% of the sequence; two of these mass peptides were located in the bacterioferritin-associated ferredoxin-like (BFD) [2Fe-2S] binding domain [32]. For mevalonate kinase, the four peptides identified spanned the domain designated mevalon_kin [33].

The proteins identified based on these analyses are listed in additional file 2, Table S1, along with their corresponding spot numbers from the 2D gel (Figure 2).

**Figure 4:**
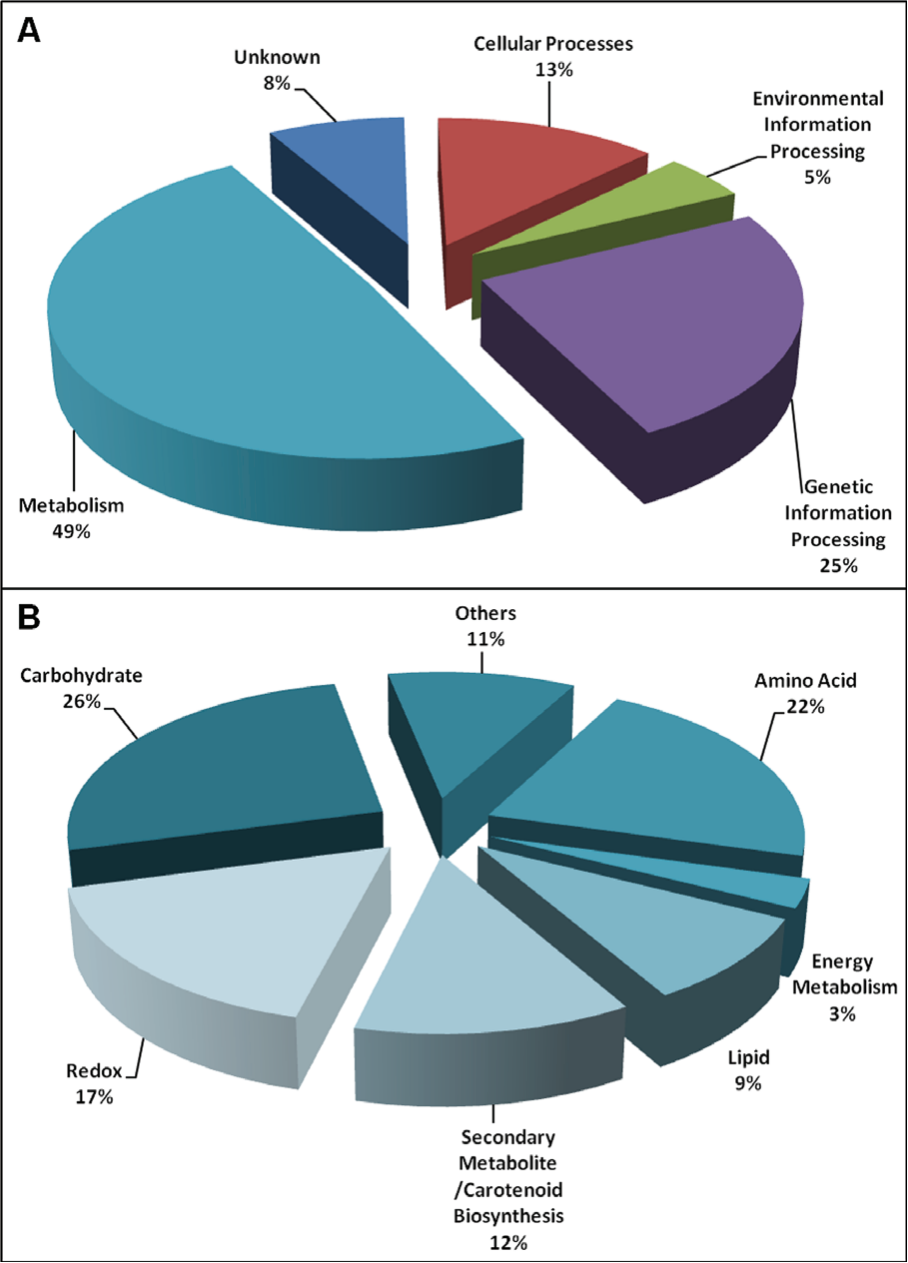
**Classification of identified proteins by cellular function**. **A**. Pie chart showing the functional classifications of the identified proteins based on annotations from the KEGG and Swiss-Prot/TrEMBL protein databases. **B**. Proteins involved in metabolism (49%) were subdivided according to pathway modules in the KEGG database. Percentages were calculated by dividing the number of proteins in the group by the total number of proteins identified and then multiplying by 100.

The proteins were classified into different groups according to their biological functions, which were determined using annotations from the KEGG database. The most abundant proteins found in this study were involved in metabolic pathways (49%; 64 proteins) (Figure 4A). Others were involved in cellular transport (17%; 13 proteins); environmental information processing, such as signal transduction proteins (6%; 5 proteins); genetic information processing including translation and transcription, replication, repair, folding and processing (25%; 33 proteins); and unknown processes (8%; 11 proteins) (Figure 4A). A similar distribution has been observed in previous yeast proteomic studies (see additional file 3, Table S2).

In the metabolism group, we identified proteins that belonged to different biosynthetic pathways, including amino acid, nucleotide, carbohydrate, energy, isoprenoid, redox and lipid metabolism (Figure 4B). The carbohydrate-related protein group, included enzymes from the glycolysis, pentose phosphate (PP) and tricarboxylic acid (TCA) pathways (see additional file 2, Table S1). In general, proteins involved in carbohydrate, amino acid, redox and lipid metabolism showed the greatest spot intensities when compared with all other identified proteins.

### Differential protein abundance during growth in MM-glucose

Statistical analyses were performed using Student's t-test (Table 1) to select spots that showed significant changes in intensity relative to the intensity in the lag phase. A total of 66 spots (corresponding to 50 proteins) showed more than two-fold changes with confidence levels of 95-99% (p < 0.05 and p < 0.01, respectively).

**Table 1:** Differentially regulated proteins of *X.dendrorhous *in MM glucose.

		^c^Normalized average spot quantity						^d^Fold change	
		
^a^SSP	^b^Description	Lag		Exponential		Stationary		E/L	S/L
		**Avg**.	SD	**Avg**.	SD	**Avg**.	SD		
	**Cellular Processes:** **Transport and motor proteins**								
6818	Putative coatomer subunit alpha	144	111	813	345	1195	155	*5.64*	8.30
8703	Myosin-associated protein	152	151	995	598	735	255	6.56	**4.84**
8711		623	441	3145	2255	2459	906	5.05	*3.95*
5719	Golgi transport protein	7637	435	2446	1101	7415	1660	-3.12	-1.03
5728		4330	676	1390	618	3494	1095	**-3.12**	-1.24
6703		9226	2086	4269	306	7877	3334	**-2.16**	-1.17
2712	SS1G_01912	13322	4086	3886	2574	5444	711	*-3.43*	*-2.45*
7403	KIP1 kinesin-related protein	1494	866	5246	2780	3349	528	3.51	*2.24*
7804	Vacuolar-sorting-associated protein 25	3952	977	11351	6299	3428	1137	**3.57**	5.03
	**Environmental Information Processing:** **Signal Transduction**								
3814	Serine/threonine-prot. phosphatase PP1-1	472	451	270	108	2273	1825	-1.75	4.81
3815		14950	1985	7701	6806	10797	2018	-5.54	1.66
3816		208	94	133	103	745	415	-1.57	3.57
5724	Nucleotide phosphodiesterase	356	91	966	339	607	196	*2.72*	1.71
0126	14-3-3. DNA damage checkpoint protein	636	515	98	102	2338	2264	-6.49	3.68
0127		261	327	236	252	3161	937	-1.11	**12.09**
0128		85	79	253	101	904	339	2.98	10.64
	**Genetic Information Processing**								
9206	Ribosomal_L15	19280	5898	6131	5697	9959	8398	*-3.14*	-1.94
7815	Mediator of RNA polymerase II	1436	1029	2487	788	3794	542	1.73	**2.64**
6707	Hypothetical protein. DNA helicase	1663	234	785	319	2342	1310	**-2.12**	1.25
6610	Replication factor C subunit 3	1663	234	785	319	2342	1310	-2.12	1.41
3228	G4P04 (Fragment)	12049	2891	7896	4292	2188	1579	-1.53	**-5.51**
4803	Calpain-like protease palB/RIM13	1155	494	1308	890	347	171	1.13	*-3.33*
		2072	391	2087	1350	1715	101	1.01	-1.21
7528	Serine/threonine protein kinase (Kin28)	1366	369	2405	840	3280	802	1.76	**2.40**
7515	Histone acetyltransferase, predicted	3162	819	10965	2273	9410	1514	*3.47*	**2.98**
7711	Cell division control protein 25, putative	957	73	2201	1398	2842	659	2.30	*2.97*
	**Metabolism**								
7407	UDP-xylose synthase	5850	468	6499	2421	12649	295	1.11	2.16
8507	ATP synthase subunit alpha	13682	2423	11233	8105	4099	3058	-1.22	**-3.34**
7801	Heat shock protein, putative	1059	268	4202	2317	2373	708	3.97	2.24
	**Lipid and Carbohydrate Metabolism**								
2523	Acetyl-CoA carboxylase	10538	888	5524	2209	10218	5489	-1.91	-1.03
2524		26474	7704	15933	13733	17308	4885	-1.66	-1.53
3516		38053	5148	12837	8209	26762	5654	**-2.96**	-1.42
7519	Phosphoglucomutase-1	1967	565	6358	1401	2562	632	**3.23**	1.30
2319	Acetyl-CoA synthetase	14327	8064	11303	10213	4218	576	-1.27	-3.40
4104	ATP-citrate synthase	18720	2582	14847	10388	11099	2402	-1.26	-1.69
4413	ATP-citrate lyase	9657	987	6925	7702	8736	2536	-1.39	-1.11
6604	Fatty acid synthase	1291	149	285	315	1978	483	*-4.52*	1.53
	**Secondary Metabolite/** **Carotenoid Biosynthesis**								
4515	Phytoene/squalene synthetase	5412	2656	13551	3057	7789	1051	**2.50**	1.44
4609	Mevalonate kinase	425	96	283	243	1246	454	-1.50	*2.93*
3517	Phosphomevalonate kinase	1005	494	270	220	367	504	*-3.72*	-2.73
6308	Diphosphomevalonate decarboxylase	2146	1521	4628	2509	5598	1347	2.16	2.61
	**Redox Metabolism**								
4401	Hypothetical oxidoreductase	6305	1432	1034	1014	1432	561	**-6.10**	**-4.40**
3606	Putative protein Cu-oxidase	741	92	184	195	1198	691	**-4.04**	1.62
5202	SDR family	2593	668	342	91	3515	418	*-7.59*	1.36
5208	Alcohol dehydrogenase	2564	1239	1008	1032	1607	578	-2.54	-1.60
4713	Monooxygenase	3930	522	4267	1706	5044	500	1.09	1.28
5703		4713	612	6594	2637	8287	916	1.40	1.76
5315	Cytochrome P450	10876	4259	16346	15386	6649	4692	1.50	-1.64
7108	Mn SOD	12020	3850	18262	13048	11032	1547	1.52	-1.09
	**Amino Acid Metabolism**								
8604	Seryl-tRNA synthetase	783	87	2517	1567	3861	203	3.21	4.93
7209	Methionyl-tRNA formyltransferase	912	290	28686	4392	17584	6195	31.44	**19.27**
7210		4348	1880	15379	2474	9085	2322	3.54	*2.09*
7816	Kynurenine 3-monooxygenase	111	73	726	424	811	64	6.56	7.33
7817		114	119	1139	751	1367	206	10.02	12.03
7819		130	84	1625	1134	1797	821	12.50	*13.82*
6821	Aspartyl-tRNA synthetase	156	81	395	76	1532	796	**2.54**	*9.84*
6828		580	11	2001	1020	2199	706	3.45	**3.79**
5410	Probable acetylornithine aminotransferase	4766	986	1794	1531	2615	447	*-2.66*	-1.82
2517	Phenylalanyl-tRNA synthetase beta chain	3325	375	813	639	2104	1397	-4.09	-1.58
5409	Glutamate dehydrogenase	2194	1506	2738	930	6893	2363	1.25	*3.14*
	**Unknown**								
2709	Conserved hypothetical protein	5609	2745	1227	889	4692	657	*-4.57*	-1.20
2710		2584	1482	1157	1630	1465	1413	-2.23	-1.76
6603	Hypothetical protein	3640	575	1014	1091	2985	120	**-3.59**	-1.22
7306	Hypothetical protein	2652	601	795	253	3569	2539	**-3.34**	1.35
6110	YALI0D17292p	10346	2105	1204	1434	8343	763	-8.59	-1.24
3503	Predicted protein	2670	367	906	897	735	650	*-2.95*	**-3.63**

^a ^SSP numbers were assigned by PDQuest software analysis.

^b ^Identifications were obtained using the Swiss-Prot and KEGG Pathways databases and contigs of *X. dendrorhous *genomic DNA.

^c ^Data derived from PDQuest estimation.

^d ^Mean fold changes compared with the 24 h cultures. Bold values indicate p < 0.01, italic p < 0.02 and underlined values indicate p < 0.05. Avg., average; SD, standard deviation.

**Figure 5:**
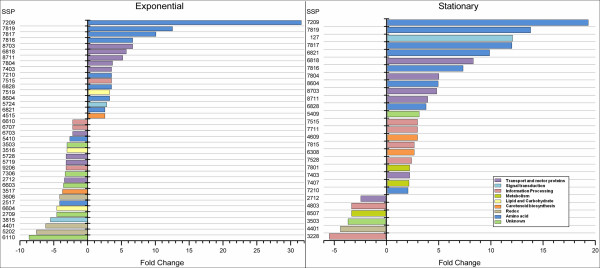
**Fold changes of differentially expressed proteins**. Proteins with more than two-fold changes (see Table 1) were plotted according to their fold change in exponential phase (left graph) or stationary phase (right graph) relative to their abundance in lag phase. The proteins are colored by their functional classification and grouped by fold change (shown on the x-axis of each graph). SSP spot identification was performed using PDQuest software.

Most of the differentially regulated proteins (63%) fell within three functional groups (metabolism, genetic information processing and cellular processes), while 13% had unknown functions (Table 1). In addition, we observed similar patterns of intensities between proteins with multiple spots, such as myosin-associated protein and Golgi transport protein (Table 1, Figure 5).

The fold-change data for proteins with differential abundances indicated that more than half of the proteins in the late exponential phase were down-regulated compared to their expression in the lag phase. In contrast most of the proteins were up-regulated in the stationary phase (Figure 5). Higher fold-changes were found for amino acid biosynthesis and transport proteins. Notably, some proteins involved in signal transduction and carotenoid biosynthesis were up-regulated in the late exponential and stationary phases. However, some redox proteins and the unknown proteins were down-regulated in both phases.

In the following sections, we present an in-depth analysis of the protein abundance patterns based on functional groups.

### Carbohydrate and lipid metabolism proteins

In the presence of glucose, the major pathways of carbohydrate metabolism are activated to produce energy for the cell. Therefore, many proteins that are important for growing cells also play a role in stationary phase growth [24]. Among these proteins, the enzymes of glycolysis and the TCA and PP pathways were identified in the 2D gels. In general, this group of proteins showed high and similar levels of abundance during growth, which is consistent with previous reports [16,34]. As indicated in Figure 5 and Table 1 only two proteins (phosphoglucomutase and acetyl-CoA carboxylase) were differentially regulated (See additional file 4, Fig. S2). It is noteworthy that these proteins not only have pivotal roles in central metabolism but are also linked to carotenogenesis.

During the induction of carotenogenesis, phosphoglucomutase (protein N°107, SSP 7519), an enzyme of the PP pathway, showed a three-fold increase in intensity (Table 1; Figure 5 and additional file 4, Fig. S2). It has been previously shown that astaxanthin synthesis requires oxygen and NADPH, which may be due to the reactions converting β-carotene to astaxanthin [15]. In addition, the PP pathway may serve as a key source of NADPH for ROS removal in response to oxidative stress [35], and phosphoglucomutase shows changes in expression related to NADPH generation when cells are treated with H_2_O_2 _[25]. Thus, our result suggests that high activity of this pathway might be required to generate sufficient NADPH for ROS quenching in *X. dendrorhous*.

Acetyl-CoA carboxylase (number SSP 3516) showed a distinct abundance pattern during growth. This protein was present at high levels during the lag phase (Figure 3A), followed by a decrease at the end of the exponential phase and then a slight increase in the stationary phase. It should be noted that only one spot showed a significant change in intensity; the other two spots showed a similar trend, although these changes were not significant (Table 1). The decrease in abundance of this protein coincided with the induction of carotenogenesis at the end of the exponential phase. This enzyme contributes to the overall control of energy metabolism in the cell and, catalyzes the carboxylation of acetyl-CoA to form malonyl-CoA, an essential substrate for fatty acid synthesis [36]. Therefore, the observed decrease in abundance might be related to the increased availability of acetyl-CoA for carotenoid biosynthesis.

Although most of the carbohydrate and lipid metabolism proteins showed similar levels during growth, we observed that several proteins related to acetyl-CoA synthesis showed maximal abundance in the lag phase, prior to the induction of carotenogenesis (Table 1), including acetyl-CoA synthetase, alcohol dehydrogenase and ATP-citrate lyase (See additional file 4, Fig. S2) [37,38]. This result indicates that carbon flux to the biosynthetic pathways, including carotenogenesis, is tightly regulated to maintain cell activity in *X. dendrorhous*.

### Redox and stress response proteins

Carotenoid accumulation is thought to be a survival strategy not only for the alga *H. pluvialis *but also for other microorganisms, including *X. dendrorhous *[39]. It has been observed that carotenoid biosynthesis in carotenoid-producing microorganisms is stimulated by oxidative stress [40,41]. Cellular antioxidant mechanisms include both non-enzymatic molecules, such as glutathione and several vitamins, and ROS scavenger enzymes, such as superoxide dismutase (SOD), catalase and glutathione peroxidase [42]. Apparently, *X. dendrorhous *lacks these enzymatic defense systems [3]; in fact, we identified only the mitochondrial MnSOD protein (see additional file 2, Table S1). This protein showed a higher abundance at the end of the exponential phase and continued to decrease during growth (Table 1 and additional file 4, Fig. S2). A proteomic study of *H. pluvialis *found that this protein is constitutively highly expressed and is progressively down-regulated after stress induction (see additional file 3, Table S2). In contrast, cytosolic CuSOD was found to be present in trace amounts and only up-regulated 48 h after stress induction [43]. Thus, an increase in the level of CuSOD and modulation of the level of MnSOD were found in response to stress in this carotenogenic alga. Moreover, in a comparative analysis of *C. albicans *grown on glucose-supplemented media, Sod21p (cytosolic manganese-dependent) was detected only in the stationary phase, whereas the Sod1p isoenzyme (Cu and Zn superoxide dismutase) was found only during exponential growth [24] (see additional file 3, Table S2). Taken together, these results suggest that the regulation of SOD is species-specific and depends on the growth phase. In the specific case of *X. dendrorhous*, we observed an increased level of MnSOD that coincided with the induction of carotenogenesis, which reinforces the antioxidant role of astaxanthin in the absence of other enzymatic antioxidant mechanisms.

For the redox and stress response proteins, we observed distinct abundance patterns occurring before or during the induction of carotenogenesis. At the end of the exponential phase, three redox proteins were more than four-fold down-regulated (Table 1, Figure 5 and additional file 4, Fig. S2), indicating that they were highly abundant in the lag phase. Interestingly, along with MnSOD, the monooxygenase and cytochrome P450 proteins were up-regulated approximately 1.5-fold at the end of the exponential phase (Table 1 and additional file 4, Fig. S2). These two proteins are closely related to the biosynthesis of many secondary metabolites, including carotenoids [22,23]. Specifically, both catalyze the addition of a single oxygen atom from molecular oxygen to a substrate and the reduction of the second oxygen atom into water, a reaction that consumes two reducing power equivalents. The final donor of electrons for the P450 monooxygenases is NADPH [44]. Moreover, CrtS (astaxanthin synthase) belongs to the cytochrome P450 protein family [45], and CpR has recently been identified as an auxiliary enzyme for CrtS during astaxanthin synthesis [46]. Two of the proteins identified in this work, cytochrome P450 and monooxygenase, could perform auxiliary reactions during astaxanthin biosynthesis; the complete identification and further characterization of these proteins is currently underway.

There are clear differences in the induction of astaxanthin synthesis between the carotenogenic microorganisms *H. pluvialis *and *X. dendrorhous*. After 24-48 h of stress induced by light and high salt, the alga undergoes morphological changes and accumulates astaxanthin for up to 12 days [43]. In the yeast, under high oxygen concentrations, astaxanthin synthesis is induced on the third day of culture, which coincides with the end of the exponential phase of growth, and allows the accumulation of astaxanthin for up to 5 days [22,23]. We found similar protein profiles for these microorganisms; however, as expected, some of the differentially regulated proteins were related to stress response and carotenogenesis. In *H. pluvialis*, the direct association between stress response and carotenogenesis is clear. For *X. dendrorhous*, during aerobic growth with a low level or the absence of the antioxidant enzymatic systems, carotenogenesis can be induced. Thus, astaxanthin could perform the antioxidant role of quenching ROS produced during cellular metabolism.

### Carotenoid biosynthetic enzymes

Using our protocol for protein extraction, we determined that 9% of all the identified proteins were membrane associated. We did not identify all of the membrane-bound enzymes that perform the late reactions of carotenogenesis, probably due to technical limitations. We have identified eight proteins related to general or specific steps of astaxanthin biosynthesis. Prenyltransferase, geranylgeranyl pyrophosphate synthase/polyprenyl synthetase, phytoene desaturase and astaxanthin synthase were present similar abundances during growth. The other four proteins showed significant fold changes (Table 1 and additional file 4, Fig. S2). First, mevalonate kinase (MK), which performs a preliminary step in IPP generation, was present at high levels in the lag phase, was down regulated in the exponential phase and was then, up regulated by approximately three-fold in the stationary phase (Table 1 Figure 5 and additional file 4, Fig. S2). This early induction is not surprising, as this enzyme performs a preliminary step in common pathways that include isoprenoid and ergosterol synthesis. In carotenogenesis, it is the second essential enzyme of the mevalonate pathway, after 3-hydroxy-3-methyl-glutaryl-CoA reductase (HMGR), which catalyzes the phosphorylation of mevalonic acid to produce phosphomevalonate. MK activity is regulated by intermediates in the pathway, such as geranyl pyrophosphate, FPP and GGPP, via feedback inhibition [47]. For phosphomevalonate kinase we observed the highest abundance at lag phase, while diphosphomevalonate decarboxylase reached its highest levels during the exponential and stationary phases. Because these two proteins perform sequential steps in the transformation of mevalonate our results indicate that this pathway is tightly regulated to ensure metabolite availability.

Another significant carotenoid-synthesis protein is phytoene/squalene synthase, which showed higher abundance at the end of the exponential growth during the induction of carotenoid synthesis (Table 1 and additional file 4, Fig. S2). This result agrees with our previously reported mRNA expression analysis, in which the maximal levels of carotenoid-specific genes were observed after three days of culture, at the end of the exponential growth phase [22,23].

In constrast, in *H. pluvialis*, the mRNA transcript levels of carotenoid-related genes reach their maximal levels 24-48 h after stress induction, and the synthesis and accumulation of astaxanthin occur 6-12 days after stress [48]. Another enzyme that performs an initial step in carotenogenesis, isopentenyl-diphosphate isomerase (IDI), shows maximum expression at 24 h after stress induction in *H. pluvialis*, and is then down-regulated as stress persist; a similar behavior has also been observed for phytoene desaturase [43,49] (see additional file 3, Table S2). Thus, carotenoid-related enzymes in both *H. pluvialis *and *X. dendrorhous *may have low turnover rates; this low rate ensures their long-term activities in astaxanthin biosynthesis.

## Conclusions

In this work, which is the first proteomic characterization of *X. dendrorhous*, we describe a protocol for the enrichment of protein extracts for membrane-bound proteins and the efficient extraction of proteins in the presence of excess hydrophobic materials such as lipids or carotenoids. We have also generated a preliminary proteome map, which will be valuable for further studies of the organism under different growth conditions.

We identified two principal types of protein regulation associated with astaxanthin biosynthesis. First, we observed a tight regulation of carbohydrate and lipid proteins, which may ensure the availability of acetyl-CoA for the cellular metabolism. Second, we observed an up-regulation of redox-specific proteins, such as monooxygenase and cytochrome P450, which likely provides the redox level necessary for the late reactions of astaxanthin synthesis.

Based on these results, it is possible to assume that the production of astaxanthin is an alternative mechanism for respondings to cellular or environmental stress conditions in *X. dendrorhous*.

Although we observed a correlation between mRNA levels and protein abundances for phytoene/squalene synthase, it will be necessary to perform a membrane proteome analysis to study the late enzymes of the astaxanthin synthesis. Moreover, detailed transcriptomic, proteomic and metabolomic studies are required to generate an integrated understanding of the biochemical, physiological and biological processes of *X. dendrorhous*, both for basic science research and for metabolic engineering applications to optimize astaxanthin production.

## Methods

### Preparation of whole-cell protein extracts

The wild type *X. dendrorhous *strain ATCC 24230 (UCD 67-385) was cultured on minimal medium with 2% glucose as a carbon source [50]. A 10-ml preculture was grown to the exponential phase (OD 6.0) at 22°C and 120 rpm. For the main culture, 250 ml of medium in a 1-L Erlenmeyer flask were inoculated with 2.5 ml of preculture and cultivated at 22°C and 120 rpm. For data analysis, triplicate cultures in the lag, late exponential and stationary growth phases were obtained (Figure 1). The cells were harvested by centrifugation at 5,000 × *g *for 10 min at 4°C. After discarding the supernatant, the pellet was washed twice with ice-cold water and centrifuged at 5,000 × *g *for 10 min at 4°C; the washed pellet was frozen in liquid nitrogen and stored at -80°C. The cell density was determined optically with a spectrophotometer at 560 nm and/or gravimetrically by measuring the cell dry weight.

Our protein extraction protocol was designed to enrich the whole-cell protein extract with membrane-bound proteins to allow for the identification of carotenogenic proteins. Yeast cells were lyophilized prior to protein extraction. After adding an equal volume around 500 μl of glass beads (500 μm) to impact-resistant 2-ml tubes, the cells were disrupted using a RiboLyzer (Hybaid-AGS, Heidelberg, Germany) for 30 s at 4.5 m/s and chilled on ice for 1 min to prevent foaming. Five-hundred microliters of lysis buffer (100 mM sodium bicarbonate, pH 8.8, 0.5% Triton × 100, 1 mM phenylmethylsulfonyl fluoride [PMSF] and protease inhibitors [Roche, Mannheim, Germany]) was then added, and the samples were incubated for 15 min on ice. Cells were disrupted five times for in a RiboLyzer for 30 s at 4.5 m/s and chilled on ice for 1 min between vortexing steps. The cell debris was removed by centrifugation at 15,000 rpm for 20 min at 4°C, and the supernatant was transferred to 1.5-ml tubes. The protein extracts then were incubated for 1 h at 4°C with a 10% v/v DNase-RNase solution (0.5 M Tris-HCl, pH 7.0, 0.5 M MgCl_2_, 100 μg/ml RNAse A [Boehringer Mannheim, Germany] and 2 μl DNase I [Boehringer Mannheim]). Next, deionized water was added to produce a final volume of 2.5 ml, and 200 μl of 0.5 M Tris (pH 6.8) and 20 μl of 1 M dithiothreitol (DTT) were added. The samples were incubated at room temperature for 30 min. Subsequently, 600 μl of water-saturated phenol was added, and the samples were mixed thoroughly and agitated at room temperature for 30 minutes. The mixture was centrifuged at 5,000 rpm at 4°C for 10 min, and the phenol phase was transferred into a fresh tube. After the addition of 20 μl of 1 M DTT and 30 μl of 8 M ammonium acetate, the samples were incubated for 30 min at room temperature. The proteins were precipitated by the addition of 2 ml of cold (-20°C) methanol and incubation over night. The precipitate was centrifuged at 13,000 rpm at 4°C for 30 min. The supernatant was discarded, and the pellet was washed twice with 70% (v/v) cold ethanol at -20°C, and incubated for 1 h at 4°C. Finally, the pellet was solubilized in 200 μl of buffer (8 M urea, 2 M thiourea, 2% [w/v] 3[(3-cholamidopropyl)dimethylammonio]-1-propanesulphonate [CHAPS], 0.01% [w/v] bromophenol blue) and stored at -80°C. The protein concentration was measured with a Bradford-based protein assay (Bio-Rad, Hercules, CA) using bovine serum albumin (BSA) as a standard.

### 2D electrophoresis

The resolubilized extract was adjusted to 500 μg in 340 μl of rehydration buffer, and 1% DTT and 2% immobilized pH gradient (IPG) buffer at pH 3-10 (IPG buffer, Amersham Biosciences, Freiburg, Germany) were added. The samples were applied to a 17-cm, non-linear pH 3-10 isoelectric focusing (IEF) strip (Immobiline DryStrip, Amersham Biosciences) and covered with mineral oil (Amersham Biosciences). IEF was carried out on a IPGphor™ system (Amersham Biosciences) using the following program:10 h at 20°C, 12 h at 30 V, 1 h at 500 V, 8 h at 1,000 V and 10 h at 8,000 V.

The strips were equilibrated for 15 min in 10 ml of equilibration solution (0.375 M Tris-HCl, pH 8.8, 6 M urea, 20% [v/v] glycerol and 2% [w/v] SDS), with 2% (w/v) DTT (reduction step), and for 15 min in 10 ml of the equilibration solution with 2% (w/v) iodoacetamide (alkylation step). The strip was then applied to a 10% SDS-PAGE gel to separate the proteins based on their molecular weights (MW). The electrophoresis conditions were 30 W per gel, applied until the bromophenol blue dye front reached the bottom of the gel.

### Protein staining and image analysis

The gels were fixed in a 10% (v/v) acetic acid and 40% (v/v) methanol solution for 2 h, stained for 3 h in a Coomassie brilliant blue (CBB) staining solution (2% [w/v] phosphoric acid, 10% [w/v] ammonium sulfate, 5% [w/v] CBB G250, 20% [v/v] methanol) and destained with 20% (v/v) methanol until the background was clear. The stained gels were scanned and analyzed with PDQuest software (version 7.1.1, Bio-Rad). The intensities of the spots were normalized to compensate for image differences generated by the experimental conditions (e.g., protein loading or staining) using the total density of the valid spots. Spot detection was performed using the PDQuest automated spot detection algorithm and checked manually. The gel image with the best protein pattern and the highest number of spots was chosen as a reference gel for image analysis, and spots in the standard gel were then matched across all gels. To compare sets of gels, the MatchSets software tool was used to calculate the mean and standard deviation of the normalized spot data. For average-fold differences in protein abundance, the normalized spot quantity from the gel at the lag growth phase was used as a reference; the relative abundance levels at later times (i.e., the late exponential and stationary phases) were calculated by dividing the normalized spot quantity in each gel by the abundance data at lag phase. Analyses were validated by Student's t-test (p < 0.05).

### MS analyses and database searches

Coomassie-stained protein spots were excised from the 2D gels and placed in 96-well plates. The spots were destained in 150 μl of 50% acetonitrile (ACN) for 5 min, in 150 μl of 50 mM NH_4_HCO_3 _and 50% ACN for 30 min, and then in 150 μl of 10 mM NH_4_HCO_3 _for 30 min while stirring at room temperature. The supernatant was removed, and the plate was dried completely at room temperature for 12 h. The proteins were digested in-gel with 15 μl of 2.5 mg/ml trypsin (Promega, Madison, WI) in 10 mM NH_4_HCO_3 _at 37°C overnight.

Samples containing the tryptic peptides were mixed 1:1 with a solution of 67:33:0.1 water: ACN: trifluoroacetic acid (TFA) (v/v) saturated with α-cyano-4-hydroxycinnamic acid (CHCA). The mass spectra were obtained with an Ultraflex MALDI-TOF-MS (Bruker, Bremen, Germany). The spectra data were analyzed in detail using FlexAnalysis software (Bruker-Daltonics). The peptide mass fingerprints generated by the MALDI-TOF MS experiments were interpreted using the Mascot search engine run on a local server (Matrix Science, London, UK). Each sample was matched to the theoretical tryptic digests of proteins from the National Center for Biotechnology Information (NCBI) non-redundant (nr) database, Swiss-Prot and MSDB. The following search parameters were set in the Mascot software: taxonomic category, fungi; no MW/pI restrictions; enzyme, trypsin; missed cleavages, 1; mass tolerance, 150 ppm and the modifications of cysteine carbamidomethylation and methionine oxidation. The database search output contained the number of matched proteins ranked according to their Mascot scores, the mass error margin and the sequence coverage of the matched peptides. A protein was only considered significant if it could be identified at least twice from the same position in independent gels, had a Mascot score higher than 50 (p < 0.05) and was the same in two of the three databases. For the contig identification, the significant Mascot score was higher than 40 (p < 0.05). All identified proteins were functionally classified according to the Kyoto Encyclopedia of Genes and Genomes (KEGG) PATHWAY database (http://www.genome.ad.jp/kegg/pathway.html).

In addition, BLAST (http://blast.ncbi.nlm.nih.gov/Blast.cgi) and CCD conserved domain (http://www.ncbi.nlm.nih.gov/Structure/cdd/cdd.shtml) searches were performed on the predicted or hypothetical proteins that had unknown functions to identify structurally and/or functionally conserved motifs.

### Carotenoid extraction and HPLC analysis

Total carotenoids were extracted from the cell pellets according to the methods described by An et al. [51]. Carotenoids were quantified by absorbance at 465 nm with an absorption coefficient of A1% = 2,100. The analyses were performed in triplicate, and pigments were normalized relative to the dry weight of the yeast.

## Competing interests

The authors declare that they have no competing interests.

## Authors' contributions

PM-M participated in the design of this study, wrote the manuscript, performed the bioinformatic analyses and carried out the protein extractions and proteomic studies. SAW participated in the set-up and standardization of the 2D-electrophoresis experiments. NK coordinated proteomics assays and critically revised the manuscript. JA collaborated in the HPLC analyses. MB participated in the statistical analyses and the initial interpretation of the results, VC conceived and coordinated this study. All authors read and approved the final manuscript.

## Supplementary Material

Additional file 1 **Fig. S1. 2D gels of soluble proteins from *X. dendrorhous *in the exponential and stationary phases of growth**. Shown are a representative 2D gels for both the exponential and stationary growth phases. Click here for file

Additional file 2 **Table S1. *X. dendrorhous *proteins identified by MALDI-TOF MS**. This table lists all MS-identified proteins that were separated by 2D electrophoresis. Click here for file

Additional file 3 **Table S2. Comparative proteomic data from yeast and the carotenogenic alga *H. pluvialis***. This table compares the most significant results from previous proteomic works on yeast and carotenogenic algae. Click here for file

Additional file 4 **Fig. S2. Differential abundance proteins from *X. dendrorhous***. Shown are a representative proteins spots during the growth. Click here for file
