# Supplementary figures and images for: Proteomic analysis of the carotenogenic yeast Xanthophyllomyces dendrorhous
Source: BMC Microbiol. 2011 Jun 13;11:131. doi: 10.1186/1471-2180-11-131 (PMC3224108; doi:10.1186/1471-2180-11-131)

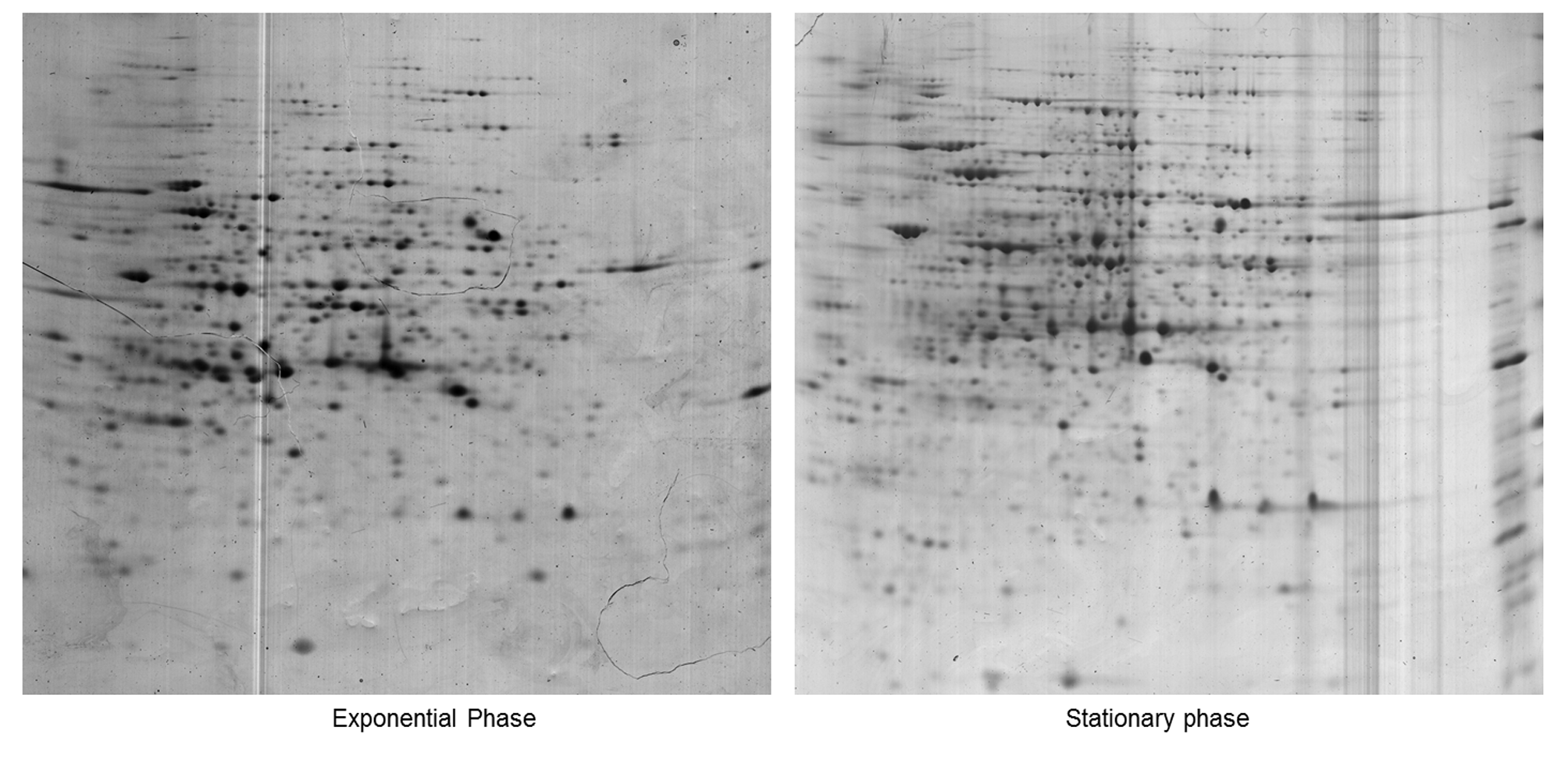

Supplement: Additional file 1 — Fig. S1. 2D gels of soluble proteins from X. dendrorhous in the exponential and stationary phases of growth. Shown are a representative 2D gels for both the exponential and stationary growth phases. [file 1471-2180-11-131-S1.TIFF]

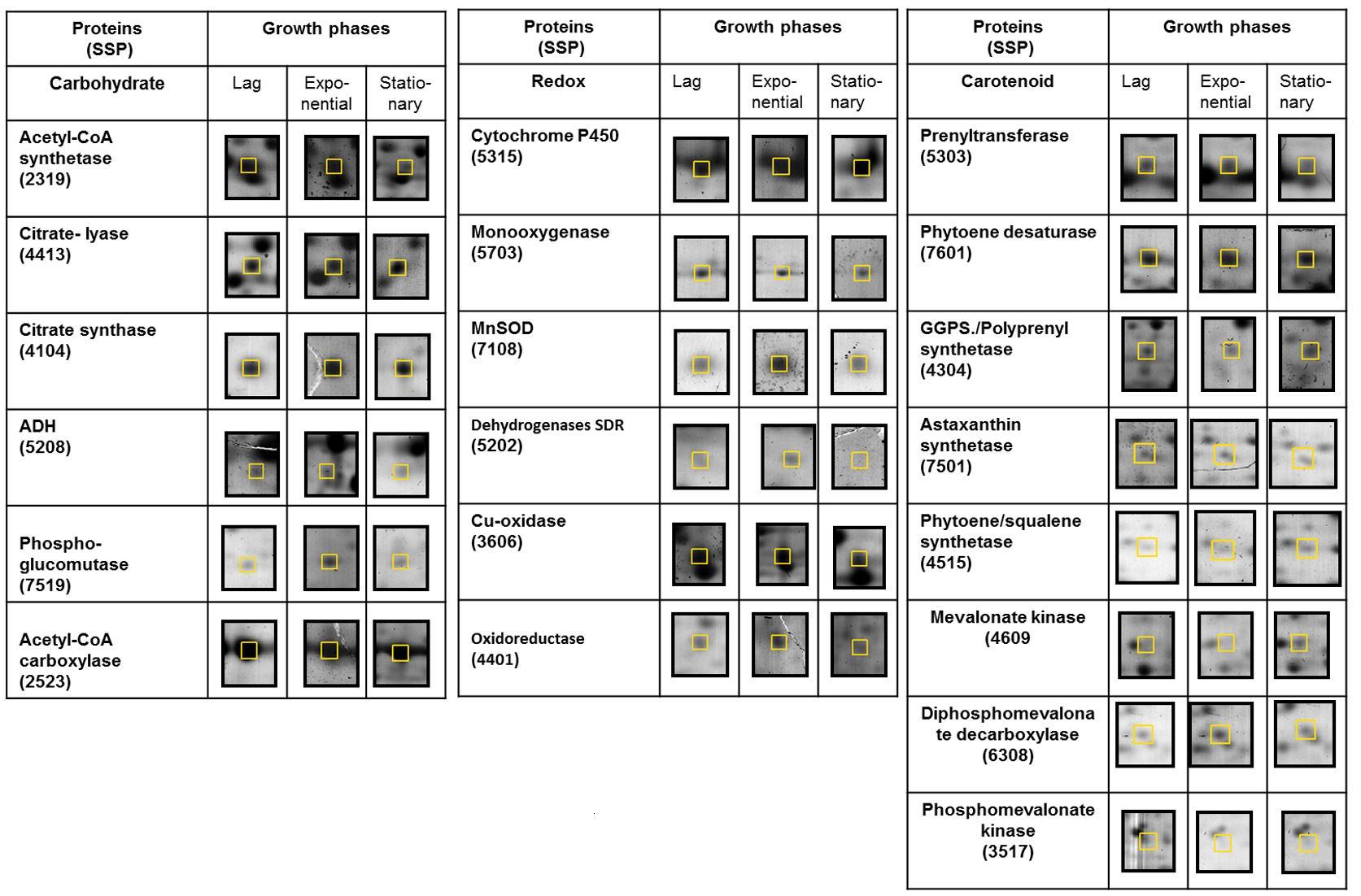

Supplement: Additional file 4 — Fig. S2. Differential abundance proteins from X. dendrorhous. Shown are a representative proteins spots during the growth. [file 1471-2180-11-131-S4.JPEG]
