# Supplementary material for: Proteomic analysis of the carotenogenic yeast Xanthophyllomyces dendrorhous
Source: BMC Microbiol. 2011 Jun 13;11:131. doi: 10.1186/1471-2180-11-131 (PMC3224108; doi:10.1186/1471-2180-11-131)
Supplement: Additional file 2 — Table S1. X. dendrorhous proteins identified by MALDI-TOF MS. This table lists all MS-identified proteins that were separated by 2D electrophoresis. [file 1471-2180-11-131-S2.DOC]

**Table S1. *X. dendrorhous* proteins identified by MALDI-TOF MS.**

| Prot N° | ID | Score | Peptide | Coverage % | Assignment | CC | Species | Spot N° | Mr/pI | |
| --- | --- | --- | --- | --- | --- | --- | --- | --- | --- | --- |
|  |  |  |  |  |  |  |  |  | Theoretical | Experimental |
|  |  |  |  |  | **Cellular Processes: Transport and Motor Proteins** |  |  |  |  |  |
| 1 | 19112071 | 54 | 5 | 5 | Putative coatomer subunit alpha | M | *Schizosaccharomyces pombe* | 6818 | 137.42/5.76 | 122.37/6.22 |
| 2 | 121792722 | 65 | 8 | 9 | Myosin -associated protein | - | *Chaetomium globosum* | 8703 | 107.26/8.01 | 107.21/7.70 |
|  |  |  |  |  |  | - |  | 8711 |  | 107.26/8.30 |
| 3 | 46099667 | 69 | 8 | 10 | Golgi transport protein | M | *Ustilago maydis* | 5719 | 124.27/6.17 | 101.09/6.07 |
|  |  |  |  |  |  | M |  | 5728 |  | 101.94/5.92 |
|  |  |  |  |  |  | M |  | 6703 |  | 100.63/6.14 |
| 4 | 66851201 | 71 | 6 | 8 | Sec1 family superfamily | - | *Aspergillus fumigatus* | 6722 | 76.29/8.03 | 86.39/6.23 |
| 5 | 154697246 | 71 | 13 | 17 | SS1G_01912 | - | *Sclerotinia sclerotiorum* | 2703 | 97.47/9.14 | 76.08/5.34 |
|  |  |  |  |  |  | - |  | 2707 |  | 76.53/5.33 |
|  |  |  |  |  |  | - |  | 2712 |  | 76.27/5.35 |
| 6 | 190407814 | 51 | 10 | 9 | ABC transporter of long-chain fatty acids | - | *Saccharomyces cerevisiae* | 6613 | 100.39/9.53 | 61.94/6.25 |
| 7 | 49650784 | 56 | 6 | 7 | KIP1 kinesin- related protein | - | *Yarrowia lipolytica* | 7403 | 109.59/8.16 | 45.58/6.39 |
|  |  |  |  |  |  | - |  | 7408 |  | 45.36/6.46 |
| 8 | 50547781 | 54 | 6 | 12 | Golgi transport component | - | *Yarrowia lipolytica* | 5520 | 78.90/5.68 | 52.33/6.03 |
| 9 | 1150540 | 145 | 10 | 46 | Actin | - | *Phaffia rhodozyma* | 3305 | 41.93/5.38 | 41.94/5.38 |
| 10 | 164426677 | 59 | 10 | 30 | Fimbrin | - | *Neurospora crassa* | 5201 | 61.50/5.77 | 34.00/5.81 |
| 11 | 83770850 | 68 | 5 | 19 | Mitochondrial oxodicarboxylate carrier | - | *Aspergillus oryzae* | 3330a | 31.32/9.96 | 40.42/5.40 |
| 12 | 74621522 | 63 | 5 | 13 | Microtubule organizing center | - | *Encephalitozoon cuniculi* | 7105 | 72.22/6.95 | 22.34/6.29 |
| 13 | 121787116 | 68 | 8 | 25 | Siderophore-iron transmembrane transporter | - | *Chaetomium globosum* | 5519 | 45.70/8.96 | 52.53/5.85 |
| 14 | 74582611 | 65 | 8 | 43 | Vacuolar protein-sorting-associated protein 25 | - | *Schizosaccharomyces pombe* | 7804 | 20.83/7.71 | 108.56/6.27 |
| 15 | 74585578 | 74 | 13 | 9 | Hypothetical protein NUM1 | - | *Candida albicans* | 8301 | 173.08/5.42 | 41.34/6.84 |
| 16 | 74693759 | 52 | 3 | 11 | AEL126Wp. Metal ion transmembrane transporter activity | - | *Ashbya gossypii* | 3330b | 43.60/5.78 | 40.42/5.40 |
| 17 | s6_c1087_p2 | 77 | 6 | 25 | Porin 3, putative | M | *X. dendrorhous* | 9210 | 30.78/9.13 | 34.40/9.70 |
|  |  |  |  |  | **Environmental Information Processing: Signal Transduction** |  |  |  |  |  |
| 18 | 226287371 | 50 | 6 | 15 | Negative regulator of the PHO system. Ser-thr kinase | - | *Paracoccidioides brasiliensis* | 5515 | 53.42/7.03 | 53.61/6.10 |
| 19 | 130710 | 65 | 4 | 15 | Serine/threonine-protein phosphatase PP1-1 | - | *Saccharomyces cerevisiae* | 3717 | 35.53/5.23 | 107.99/5.42 |
|  |  |  |  |  |  | - |  | 3718 |  | 108.00/5.39 |
|  |  |  |  |  |  | - |  | 3808 |  | 108.15/5.49 |
|  |  |  |  |  |  | - |  | 3814 |  | 108.14/5.52 |
|  |  |  |  |  |  | - |  | 3815 |  | 108.11/5.45 |
|  |  |  |  |  |  | - |  | 3816 |  | 108.19/5.38 |
| 20 | 74626964 | 68 | 7 | 14 | Nucleotide phosphodiesterase | - | *Candida albicans* | 5724 | 66.07/5.87 | 79.30/6.10 |
| 21 | 74665510 | 68 | 5 | 21 | 14-3-3. DNA damage checkpoint protein | - | *Lentinula edodes* | 0126 | 29.00/4.67 | 29.20/4.85 |
|  |  |  |  |  |  | - |  | 0127 |  | 29.10/4.56 |
|  |  |  |  |  |  | - |  | 0128 |  | 29.00/4.30 |
| 22 | 74670489 | 68 | 12 | 9 | GTPase activating protein | - | *Aspergillus fumigatus* | 8502 | 142.88/7.38 | 59.96/6.90 |
| 23 | 74683137 | 58 | 10 | 17 | Protein kinase | - | *Cryptococcus neoformans* | 2321 | 64.11/5.40 | 40.04/5.35 |
|  |  |  |  |  | **Genetic Information Processing:** |  |  |  |  |  |
|  |  |  |  |  | **Translation and Translation Regulation** |  |  |  |  |  |
| 24 | 121787153 | 62 | 6 | 28 | Ribosomal_L15 | - | *Chaetomium globosum* | 9206 | 32.84/10.21 | 35.60/10.10 |
| 25 | 74587115 | 57 | 10 | 20 | 60S ribosomal protein L5 | - | *Candida albicans* | 7304 | 34.48/7.08 | 40.18/6.38 |
| 26 | 74603032 | 73 | 8 | 26 | Protein PXR1 | - | *Debaryomyces hansenii* | 8104 | 37.01/9.87 | 22.46/7.28 |
| 27 | 119397404 | 51 | 6 | 16 | Eukaryotic translation initiation factor 3 subunit H | - | *Aspergillus clavatus* | 5417 | 41.26/5.96 | 47.08/5.81 |
| 28 | 74702348 | 60 | 4 | 32 | RNase H | - | *Ustilago maydis* | 2215 | 13.00/10.18 | 32.26/5.33 |
| 29 | 73918962 | 60 | 6 | 26 | Pre-mRNA-splicing factor | - | *Schizosaccharomyces pombe* | 4411 | 21.41/6.85 | 44.66/5.76 |
| 30 | 417834 | 52 | 5 | 8 | SWI/SNF complex subunit SWI3 | - | *Saccharomyces cerevisiae* | 4810 | 92.87/4.78 | 111.94/5.72 |
| 31 | 158564282 | 62 | 10 | 11 | Mediator of RNA polymerase II transcription subunit 14 | - | *Aspergillus oryzae* | 7815 | 123.92/9.01 | 109.01/6.40 |
| 32 | 121791431 | 78 | 7 | 17 | Hypothetical protein. DNA helicase | - | *Chaetomium globosum* | 5717 | 67.64/5.88 | 76.77/6.07 |
|  |  |  |  |  |  | - |  | 6701 |  | 76.24/6.12 |
|  |  |  |  |  |  | - |  | 6707 |  | 77.06/6.20 |
| 33 | 160380696 | 52 | 4 | 8 | ATP-dependent RNA helicase dbp9 | - | *Botryotinia fuckeliana* | 3613 | 67.51/9.16 | 64.04/5.50 |
| 34 | 74692018 | 50 | 8 | 13 | AGL075Cp. Regulation of transcription | - | *Ashbya gossypii* | 4608 | 67.50/8.17 | 66.05/5.75 |
| 35 | s5_c2133_p | 44 | 8 | 7 | General RNA polymerase II transcription factor | - | *X. dendrorhous* | 4812 | 133.93/5.88 | 118.50/5.60 |
|  |  |  |  |  | **Genetic Information Processing Replication and Repair** |  |  |  |  |  |
| 36 | 74676175 | 69 | 12 | 24 | DNA polymerase alpha-associated DNA helicase A | - | *Schizosaccharomyces pombe* | 8503 | 75.35/6.74 | 52.83/7.11 |
| 37 | 74630069 | 70 | 8 | 11 | DNA replication helicase | - | *Encephalitozoon cuniculi* | 8304 | 108.05/5.87 | 41.38/7.19 |
| 38 | 585844 | 61 | 8 | 24 | Replication factor C subunit 3 | - | *Saccharomyces cerevisiae* | 6610 | 38.46/6.07 | 67.09/6.21 |
| 39 | 48474713 | 50 | 7 | 13 | Meiotically up-regulated gene 72 protein | - | *Schizosaccharomyces pombe* | 4408 | 62.80/8.92 | 43.63/5.68 |
| 40 | 74583120 | 51 | 7 | 36 | G4P04 (Fragment) | - | *Emericella nidulans* | 3228 | 24.32/8.97 | 34.64/5.44 |
| 41 | 121782353 | 87 | 12 | 17 | Predicted protein. Exonuclease V, alpha subunit | - | *Chaetomium globosum* | 9209 | 43.71/9.62 | 35.39/9.44 |
| 42 | 74687723 | 55 | 6 | 24 | Ribonuclease H, putative | - | *Cryptococcus neoformans* | 8001 | 35.55/5.85 | 17.80/6.60 |
| 43 | s6_c1236_p | 43 | 12 | 12 | DNA topoisomerase II, putative | - | *X. dendrorhous* | 6811 | 166.80/8.09 | 118.80/6.27 |
| 44 | s6_c1477_p1 | 41 | 6 | 15 | Cell division control protein 25, putative | M | *X. dendrorhous* | 7711 | 75.20/9.43 | 72.30/6.38 |
| 45 | s6_c7188_p1 | 43 | 10 | 14 | Ku80-like protein | - | *X. dendrorhous* | 4725 | 94.60/5.10 | 105.50/5.70 |
| 46 | s5_c9012_p | 41 | 9 | 8 | DNA ATP-dependent helicase | - | *X. dendrorhous* | 5607 | 127.47/6.61 | 67.20/6.10 |
| 47 | s5_c863_p | 48 | 14 | 10 | DNA-directed RNA polymerase I | - | *X. dendrorhous* | 7810 | 182.28/6.26 | 129.50/6.30 |
| 48 | s5_c6133_p | 47 | 12 | 12 | DNA polymerase gamma, predicted | - | *X. dendrorhous* | 8506 | 61.88/9.51 | 57.70/8.20 |
| 49 | s6_c11128_p | 41 | 13 | 9 | Histone acetyltransferase, predicted | - | *X. dendrorhous* | 7515 | 164.57/6.71 | 61.00/6.40 |
|  |  |  |  |  | **Genetic Information Processing** |  |  |  |  |  |
| 50 | 73921582 | 51 | 5 | 11 | Calpain-like protease palB/RIM13 | - | *Aspergillus oryzae* | 4803 | 96.09/5.93 | 106.07/5.62 |
|  |  |  |  |  |  | - |  | 4806 |  | 105.94/5.91 |
| 51 | 74700737 | 51 | 6 | 13 | Hypothetical protein. ATP-binding, Chaperone | - | *Ustilago maydis* | 5602 | 60.13/5.71 | 61.65/5.91 |
| 52 | 74668029 | 51 | 5 | 8 | Vacuolar aspartyl aminopeptidase Lap4, putative | - | *Sartorya fumigata* | 5316 | 56.33/6.61 | 40.00/6.00 |
| 53 | 74668502 | 61 | 4 | 18 | Serine/threonine protein kinase (Kin28), putative | - | *Aspergillus fumigatus* | 7528 | 45.22/6.03 | 58.74/6.53 |
| 54 | 74589431 | 56 | 5 | 10 | Likely 26S proteasome regulatory particle subunit Rpn6p | - | *Candida albicans* | 5601a | 49.52/5.85 | 62.80/5.89 |
| 55 | 74681797 | 60 | 13 | 18 | Ubiquitin-protein ligase | - | *Cryptococcus neoformans* | 7510a | 88.93/7.92 | 61.20/6.30 |
| 56 | s6_c1168_p | 47 | 11 | 13 | Cell polarity protein, putative | M | *X. dendrorhous* | 3805 | 172.79/5.93 | 115.10/5.40 |
|  |  |  |  |  | **Metabolism: Redox** |  |  |  |  |  |
| 57 | 119413925 | 68 | 9 | 23 | Monooxygenase, putative | - | *Neosartorya fischeri* | 4713 | 49.01/6.32 | 87.24/5.80 |
|  |  |  |  |  |  | - |  | 5703 |  | 87.14/5.91 |
| 58 | 121792603 | 60 | 7 | 23 | Hypothetical protein. Oxidoreductase activity | - | *Chaetomium globosum* | 4401 | 35.99/9.41 | 43.95/5.54 |
| 59 | 74680537 | 51 | 5 | 8 | Hypothetical protein. Uncharacterized protein Cu-oxidase | - | *Emericella nidulans* | 3606 | 76.11/5.4 | 67.83/5.39 |
| 60 | 33313426 | 135 | 11 | 41 | Alcohol dehydrogenase | - | *Phaffia rhodozyma* | 5208 | 39.06/6.07 | 39.06/6.07 |
| 61 | 121797391 | 50 | 6 | 23 | Short-chain dehydrogenase/reductase (SDR) family | - | *Aspergillus oryzae* | 5202 | 27.70/5.57 | 37.47/5.87 |
| 62 | 14134949 | 50 | 4 | 15 | Sequence 6 from Patent EP1111067. MnSOD | - | *Phaffia rhodozyma* | 7108 | 22.12/6.43 | 22.12/6.43 |
| 63 | 121800584 | 52 | 5 | 21 | Electron transfer flavoprotein | - | *Aspergillus oryzae* | 3112 | 28.23/8.98 | 30.32/5.49 |
| 64 | 74680498 | 68 | 6 | 18 | Hypothetical protein. Cytochrome P450 | M | *Emericella nidulans* | 5315 | 52.86/8.61 | 39.80/6.07 |
| 65 | 927720 | 58 | 4 | 30 | Peroxiredoxin TSA2 | - | *Saccharomyces cerevisiae* | 8205 | 21.71/6.74 | 30.37/7.91 |
| 66 | 74632761 | 56 | 5 | 16 | YALI0F09097p. Potential oxidoreductase | - | *Yarrowia lipolytica* | 4310 | 38.16/5.36 | 40.06/5.54 |
|  |  |  |  |  | **Metabolism: Amino Acid** |  |  |  |  |  |
| 67 | 74625934 | 59 | 6 | 12 | Seryl-tRNA synthetase, mitochondrial | - | *Schizosaccharomyces pombe* | 8604 | 51.69/8.68 | 59.52/7.06 |
| 68 | 151942753 | 57 | 10 | 10 | Mitochondrial isoleucyl-tRNA synthetase | - | *Saccharomyces cerevisiae* | 7805 | 116.57/8.43 | 116.89/6.28 |
|  |  |  |  |  |  | - |  | 7808 |  | 116.65/6.30 |
|  |  |  |  |  |  | - |  | 7811 |  | 116.57/6.37 |
| 69 | 121928306 | 72 | 4 | 30 | Methionyl-tRNA formyltransferase | - | *Aspergillus oryzae* | 7209 | 28.83/9.59 | 36.14/6.28 |
|  |  |  |  |  |  | - |  | 7210 |  | 36.36/6.30 |
| 70 | 223635247 | 57 | 6 | 12 | Kynurenine 3-monooxygenase | M | *Pichia stipitis* | 7816 | 54.63/6.95 | 120.07/6.41 |
|  |  |  |  |  |  | M |  | 7817 |  | 120.06/6.43 |
|  |  |  |  |  |  | M |  | 7819 |  | 120.08/6.53 |
| 71 | 119413553 | 70 | 6 | 8 | Aspartyl-tRNA synthetase, cytoplasmic | - | *Neosartorya fischeri* | 6821 | 108.47/6.46 | 108.74/6.23 |
|  |  |  |  |  |  | - |  | 6828 |  | 108.62/6.25 |
| 72 | 30913511 | 51 | 6 | 16 | tRNA (cytosine-5-)-methyltransferase ncl1 | - | *Schizosaccharomyces pombe* | 3511 | 45.21/6.47 | 56.97/5.48 |
| 73 | 30912640 | 60 | 5 | 13 | Probable acetylornithine aminotransferase | - | *Schizosaccharomyces pombe* | 5410 | 48.29/8.75 | 45.45/6.09 |
| 74 | 12229964 | 57 | 7 | 12 | Phenylalanyl-tRNA synthetase beta chain | - | *Schizosaccharomyces pombe* | 2517 | 67.71/5.29 | 57.05/5.36 |
| 75 | 74624697 | 50 | 2 | 66 | Histidine biosynthesis trifunctional protein | - | *Saccharomyces bayanus* | 4003 | 5.72/4.29 | 10.60/5.60 |
| 76 | 74696766 | 61 | 6 | 20 | Methyltransferase protein | - | *Neurospora crassa* | 6106 | 31.78/5.56 | 25.68/6.25 |
| 77 | 121814778 | 52 | 3 | 9 | Acetylglutamate kinase | - | *Gibberella zeae* | 3330c | 44.36/8.74 | 40.42/5.40 |
| 78 | 74615252 | 76 | 11 | 20 | NCU09184.1. Indoleamine dioxygenase | - | *Neurospora crassa* | 7510b | 74.42/6.99 | 61.20/6.30 |
| 79 | 209406130 | 98 | 10 | 30 | Glutamate dehydrogenase | - | *X. dendrorhous* | 5409 | 48.98/5.99 | 46.79/6.07 |
| 80 | s6_c1090_p2 | 52 | 8 | 20 | Phospho-2-dehydro-3-deoxyheptonate aldolase | - | *X. dendrorhous* | 3225 | 41.29/6.42 | 35.70/5.40 |
|  |  |  |  |  | **Metabolism: Energy Metabolism** |  |  |  |  |  |
| 81 | 74684619 | 73 | 7 | 13 | ATP synthase subunit alpha | M | *Cryptococcus neoformans* | 8507b | 58.17/9.01 | 58.20/9.01 |
| 82 | 74701945 | 156 | 18 | 46 | ATP synthase subunit beta | M | *Ustilago maydis* | 1504 | 55.69/5.28 | 55.66/5.31 |
|  |  |  |  |  |  | M |  | 1505 |  | 55.03/5.30 |
|  |  |  |  |  |  | M |  | 1506 |  | 55.33/5.29 |
|  |  |  |  |  |  | M |  | 1507 |  | 55.72/5.28 |
|  |  |  |  |  | **Metabolism: Lipid** |  |  |  |  |  |
| 83 | 121803722 | 50 | 3 | 18 | Phosphatidylinositol synthase | M | *Aspergillus oryzae* | 3331 | 30.81/8.49 | 41.40/5.49 |
| 84 | 46426876 | 57 | 14 | 8 | CQ798506 NID. Acetyl-CoA carboxylase, cytosolic | - | *Phaffia rhodozyma* | 2524 | 245.73/5.82 | 52.04/5.37 |
|  |  |  |  |  |  | - |  | 2523 |  | 52.73/5.35 |
|  |  |  |  |  |  | - |  | 3516 |  | 51.28/5.41 |
| 85 | 74672173 | 53 | 7 | 16 | Phosphatidylserine decarboxylase, putative | - | *Aspergillus fumigatus* | 6511 | 61.26/9.45 | 57.10/6.24 |
| 86 | 74694301 | 68 | 10 | 17 | ADR052Wp. Similar acyl-CoA synthetase | M | *Ashbya gossypii* | 4603 | 84.26/7.49 | 63.56/5.60 |
| 87 | s6_c1131_p | 83 | 7 | 23 | Acetyl-CoA C-acetyltransferase | - | *X. dendrorhous* | 6304 | 45.54/8.38 | 41.30/6.20 |
| 88 | s6_c7358_p | 47 | 11 | 8 | Fatty acid synthase | - | *X. dendrorhous* | 6604 | 223.28/6.15 | 63.60/6.20 |
|  |  |  |  |  | **Metabolism: Secondary metabolite/carotenoid biosynthesis** |  |  |  |  |  |
| 89 | 46111687 | 56 | 4 | 14 | Hypothetical protein. Phytoene/squalene synthetase | - | *Gibberella zeae* | 4515 | 52.28/9.11 | 52.82/5.66 |
| 90 | 125407 | 51 | 4 | 15 | Mevalonate kinase | - | *Saccharomyces cerevisiae* | 4609 | 48.94/5.36 | 63.47/5.77 |
| 91 | 74671624 | 55 | 6 | 15 | Prenyltransferase, putative | - | *Aspergillus fumigatus* | 5303 | 44.86/5.66 | 40.47/5.91 |
| 92 | 74589056 | 61 | 5 | 14 | Protein BTS1. GGPS./Polyprenyl synthetase | - | *Candida albicans* | 4304 | 38.28/5.48 | 41.28/5.62 |
| 93 | 33465817 | 44 | 5 | 10 | Phytoene desaturase | - | *X. dendrorhous* | 7601 | 65.06/6.17 | 61.30/6.3 |
| 94 | 323710252 | 41 | 5 | 10 | Mutant astaxanthin synthetase | M | *X. dendrorhous* | 7501 | 62.61/6.29 | 53.3/6.30 |
| 95 | PM1619_p | 49 | 11 | 20 | Diphosphomevalonate decarboxylase, predicted | - | *X. dendrorhous* | 6308 | 43.41/575 | 41.80/6.20 |
| 96 | PM41-401_p | 43 | 4 | 9 | Phosphomevalonate kinase | - | *X. dendrorhous* | 3517 | 57.59/6.03 | 59.4/5.50 |
|  |  |  |  |  | **Metabolism** |  |  |  |  |  |
| 97 | 238054304 | 68 | 14 | 16 | Nitrite reductase [NAD(P)H] | - | *Emericella nidulans* | 3603 | 123.99/5.81 | 66.24/5.38 |
| 98 | 74627095 | 56 | 8 | 17 | Heat shock protein 70 | - | *Cryptococcus curvatus* | O711 | 70.22/4.98 | 74.69/4.42 |
| 99 | 74686470 | 76 | 6 | 12 | Heat shock protein 70 | - | *Cryptococcus neoformans* | 3701 | 69.53/4.94 | 69.53/5.40 |
|  |  |  |  |  |  | - |  | 3715 |  | 69.49/5.51 |
|  |  |  |  |  |  | - |  | 3716 |  | 69.37/5.45 |
| 100 | 74612899 | 73 | 9 | 19 | UDP-xylose synthase | - | *Cryptococcus neoformans* | 7407 | 46.75/6.12 | 47.50/6.45 |
| 101 | 121794239 | 72 | 8 | 25 | 1-aminocyclopropane-1-carboxylate deaminase | - | *Magnaporthe grisea* | 5604 | 42.12/8.65 | 61.62/6.05 |
|  |  |  |  |  |  |  |  | 6608 |  | 61.49/6.18 |
| 102 | 74666885 | 55 | 4 | 9 | FMN-dependent dehydrogenase family protein | - | *Aspergillus fumigatus* | 3330d | 41.31/7.03 | 40.42/5.40 |
| 103 | s6_c1356_p | 41 | 9 | 11 | Heat shock protein, putative | - | *X. dendrorhous* | 7801 | 108.35/6.48 | 115.00/6.30 |
|  |  |  |  |  | **Metabolism: Carbohydrate** |  |  |  |  |  |
| 104 | 121780797 | 62 | 10 | 26 | Similar to acetyl-CoA synthetase | - | *Chaetomium globosum* | 2319 | 60.08/6.33 | 39.71/5.37 |
| 105 | 74702395 | 54 | 5 | 5 | Alpha-glucosidase | - | *Ustilago maydis* | 2614 | 116.71/5.68 | 66.94/5.36 |
|  |  |  |  |  |  | - |  | 2619 |  | 67.14/5.33 |
|  |  |  |  |  |  | - |  | 2621 |  | 66.82/5.35 |
|  |  |  |  |  |  | - |  | 2622 |  | 67.05/5.34 |
|  |  |  |  |  |  | - |  | 2623 |  | 67.16/5.34 |
| 106 | 119409891 | 60 | 7 | 15 | Melibiase, putative | - | *Neosartorya fischeri* | 7625 | 80.84/5.84 | 63.12/6.39 |
| 107 | 464368 | 60 | 7 | 14 | Phosphoglucomutase-1 | - | *Saccharomyces cerevisiae* | 7519 | 63.47/6.82 | 56.03/6.39 |
| 108 | 74692484 | 68 | 9 | 12 | Phosphorylase | - | *Ashbya gossypii* | 5605 | 102.84/5.44 | 62.63/6.08 |
| 109 | 74637885 | 58 | 6 | 12 | Neutral trehalase | - | *Candida glabrata* | 2309 | 86.80/6.54 | 40.53/5.36 |
| 110 | 74705139 | 52 | 6 | 12 | Glucokinase | - | *Trichoderma reesei* | 6609 | 59.94/5.26 | 62.39/6.21 |
| 111 | 121807791 | 55 | 3 | 13 | Phosphoglycerate kinase | - | *Rhizopus oryzae* | 4201 | 44.75/6.29 | 34.09/5.53 |
| 112 | 3122121 | 90 | 11 | 35 | Glyceraldehyde-3-phosphate dehydrogenase | - | *Phaffia rhodozyma* | 4312 | 36.33/5.79 | 40.10/5.79 |
|  |  |  |  |  |  | - |  | 5314 |  | 40.00/6.10 |
| 113 | 74670647 | 50 | 6 | 14 | ATP-citrate lyase subunit (Acl) | - | *Aspergillus fumigatus* | 4413 | 52.99/5.88 | 48.76/5.77 |
| 114 | 30912748 | 65 | 6 | 14 | Probable ATP-citrate synthase subunit 1 | - | *Schizosaccharomyces pombe* | 4104 | 67.84/8.11 | 29.12/5.58 |
| 115 | 74631399 | 52 | 6 | 13 | Transaldolase | - | *Debaryomyces hansenii* | 2213 | 35.99/5.23 | 39.33/5.37 |
| 116 | 121808353 | 51 | 5 | 12 | Beta-fructosidase (Fragment) | - | *Saccharomyces cariocanus* | 5601b | 58.64/4.56 | 62.80/5.89 |
| 117 | s5_c1234_p2 | 41 | 5 | 12 | NAD-dependent formate dehydrogenase | - | *X. dendrorhous* | 7312 | 40.22/6.38 | 39.80/6.40 |
| 118 | s6_c1550_p2 | 112 | 11 | 45 | NAD-malate dehydrogenase | - | *X. dendrorhous* | 3206 | 34.83./5.18 | 36.10/5.4 |
| 119 | s6_c11170_p | 56 | 8 | 7 | Pyruvate carboxylase | - | *X. dendrorhous* | 6809 | 130.84/5.93 | 124.80/6.20 |
| 120 | s6_c1090_p1 | 42 | 4 | 10 | Oxoglutarate dehydrogenase, predicted | - | *X. dendrorhous* | 7532 | 56.40/6.11 | 52.50/6.30 |
|  |  |  |  |  | **Unknown** |  |  |  |  |  |
| 121 | 74669512 | 65 | 9 | 14 | Conserved hypothetical protein | - | *Aspergillus fumigatus* | 2709 | 90.26/9.33 | 73.18/5.33 |
|  |  |  |  |  |  | - |  | 2710 |  | 74.25/5.33 |
|  |  |  |  |  |  | - |  | 2711 |  | 73.10/5.34 |
| 122 | 121778823 | 70 | 9 | 21 | Hypothetical protein | - | *Chaetomium globosum* | 6603 | 48.78/9.74 | 67.15/6.14 |
| 123 | 121779248 | 68 | 10 | 18 | Hypothetical protein | - | *Chaetomium globosum* | 6601 | 69.86/7.23 | 65.31/6.12 |
| 124 | 74693118 | 68 | 7 | 8 | AFL122Wp | - | *Ashbya gossypii* | 4422 | 57.74/10.06 | 47.08/5.71 |
|  |  |  |  |  |  | - |  | 5416a |  | 46.90/5.98 |
| 125 | 74700237 | 60 | 5 | 25 | Hypothetical protein | - | *Ustilago maydis* | 7306 | 31.64/8.67 | 39.91/6.51 |
| 126 | 74634458 | 50 | 6 | 36 | YALI0D17292p . Similarity (Fragment) | - | *Yarrowia lipolytica* | 6110 | 16.90/7.00 | 22.36/6.16 |
| 127 | 121754798 | 52 | 5 | 9 | Hypothetical protein. | - | *Coccidioides immitis* | 6314 | 57.50/6.78 | 39.66/6.17 |
|  |  |  |  |  |  | - |  | 6317 |  | 39.80/6.22 |
| 128 | 121800089 | 52 | 4 | 17 | Predicted protein | - | *Aspergillus oryzae* | 3503 | 43.71/4.46 | 60.54/5.40 |
| 129 | 119484962 | 67 | 6 | 12 | C6 zinc finger domain protein | - | *Neosartorya fischeri* | 5416b | 50.36/8.95 | 46.90/5.98 |
| 130 | 169766802 | 54 | 5 | 35 | Predicted protein | - | *Aspergillus oryzae* | O310b | 21.41/5.23 | 41.40/4.20 |
| 131 | s6_c1795_p1 | 62 | 12 | 18 | Hypothetical protein | - | *X. dendrorhous* | 7508 | 56.60/6.30 | 65.45/5.09 |

All identified non-redundant proteins were manually assigned N°s. The IDs correspond to the accession numbers from the NCBI database and contigs of *X. dendrorhous*. The score, peptide and coverage values correspond to the data from the MASCOT software analysis. Assignments were made according to Swiss-Prot and KEGG. CC corresponds to cellular component localization. The spot N° data were generated by PDQuest software. Theoretical Mr/pI data were obtained from the MASCOT protein identification, and experimental Mr/pI data were calculated by PDQuest. Mr, molecular mass; pI, isoelectric point. Capital letters in spot N°s; indicate overlapping spots.
