# Supplementary material for: Proteomic analysis of the carotenogenic yeast Xanthophyllomyces dendrorhous
Source: BMC Microbiol. 2011 Jun 13;11:131. doi: 10.1186/1471-2180-11-131 (PMC3224108; doi:10.1186/1471-2180-11-131)
Supplement: Additional file 3 — Table S2. Comparative proteomic data from yeast and the carotenogenic alga H. pluvialis. This table compares the most significant results from previous proteomic works on yeast and carotenogenic algae. [file 1471-2180-11-131-S3.DOC]

**Supplementary Table 2. Comparison of proteomic data from yeast and the carotenogenic alga *H. pluvialis.***

| Ref. | Growth/stain conditions | Identification | Organism | Proteins analized | Functional classification | Principal observations |
| --- | --- | --- | --- | --- | --- | --- |
| * | Rich medium, mid-exponential phase  / silver stained | 2D gel  strip 3-10  MALDI-TOFMS  and/or nanoLC-MS/MS | *S. pombe* | -Total proteins observed: 1500  -Protein analized:157/364 | - Metabolic protein (40.1 %)  -Synthesis and transcription (13.4%)  -Protein folding, modification and targeting (9.6%)  -Cellular transport (8.9%)  -Cell rescue, defense and stress (7.0%)  -Cell organization and biogenesis (3.8%)  -Cell cycle (2.5%)  -Unknown 14.6% | - Several metabolic enzymes are found in multiple spots. (enolase 1–1, glyceraldehyde-3-phosphate dehydrogenase 1, fructose-bisphosphate aldolase, phosphoglycerate mutase, phosphoglycerate kinase, triosephosphate  isomerase, and alcohol dehydrogenase.  -In this study, 58 different proteins (among 157 different proteins) were found in multiple spots. The presence of these multiple spots on 2-D gels was considered to be due to PTM or proteolytic processing. |
| 43 | Stress conditions (0,30 min, 12 h,24 h,48 h, or 72 h)/Coomassie Blue | 2D gel  strip 3-10  MALDI–TOF  MS | *H. pluvialis* | -Total proteins observed: 900; analyzed 70 | -13 proteins were down-regulated (some photosynthesis and nitrogen assimilation, TCA cycle proteins).  -19 were up-regulated (SOD and HSP, glycolysis).  -8 proteins were transiently down-regulated, followed by up-regulation.  -30 proteins were transiently up-regulated, followed by down-regulation. (some mitochondrial respiratory proteins and stress proteins).  These proteins were involved in a broad range of functions, including cellular processes and stress responses; central and secondary metabolism; energy metabolism; biosynthesis of fatty acids, carbohydrates, carotenoids and amino acids; and protein translation, transport and binding. | -Cellular accumulation of the antioxidant astaxanthin occurred after initiation of oxidative stress and reached its maximum cellular level after six or more days of stress.  **-Down-regulated:** glutamine synthetase, ubiquitin-activating enzyme, aconitate hydratase, nitrite reductase [NAD(P)H], ferredoxin-nitrite reductase, pyruvate kinase, phosphoglycerate kinase, superoxide dismutase [Mn/Fe].  **-Up-regulated:** glucose-6-phosphate isomerase, aldolase, triose phosphate isomerase, glyceraldehyde-3-phosphate dehydrogenase, phosphoglycerate kinase, enolase, lactate dehydrogenase, alcohol dehydrogenase, phosphoenolpyruvate carboxylase, malate dehydrogenase, glucose-6-phosphate 1-dehydrogenase, 6-phosphogluconate dehydrogenase, transaldolase, actin, 14-3-3-like protein, triosephosphate isomerase, Heat-shock 22 kDa protein-like, alcohol dehydrogenase 2, glutathione S-transferase, ferredoxin NADP+ reductase, thioredoxin, ubiquitin.  **-Proteins transiently down-regulated, then up-regulated:** Protein kinase homolog, putative alcohol oxidase, ATP synthase β-subunit, ATP synthase α-subunit.  **-Proteins transiently up-regulated, then down-regulated:** Heat-shock protein 81-1 (HSP90), dnaK (chaperone HSP70), ATPase β-chain, mitocondrial, vacuolar ATPase subunit α, 6-Phosphogluconate dehydrogenase, tubulin b-1/ b-2 chain, catalase, enolase, glucose-6-phosphate isomerase, phosphoglycerate kinase, peroxidase, transaldolase, glyceraldehyde-3-phosphate dehydrogenase, isopentenyl-pyrophosphate isomerase, phosphatase PP1, superoxide dismutase [Cu–Zn], NADH-plastoquinone oxidoreductase. |
| ** | Rich medium, mid-exponential phase  was subjected to oxidative stress H_2_O_2_/DIGE | 2D gel  strip 4-7  MALDI-MS | *S. pombe* | -Total proteins observed: ND; analyzed : 110 | Proteins were involved in a broad range of functions, including cellular processes and stress response; central and secondary metabolism; energy metabolism; biosynthesis of fatty acids, carbohydrates, carotenoids and amino acids; and protein translation, transport and binding. | **Down-regulated (51/110 proteins):** ATPase, ubiquitin-activating enzyme, 3-isopropylmalate dehydratase, HSP70, HSP90, DNAK-type molecular, V-type ATPase, t-complex protein 1, phosphoglucomutase, precursor of GMP synthase, malate oxidoreductase [NAD], glucose-6-phosphate dehydrogenase, malate oxidoreductase, hexokinase, mitochondrial F1-ATPase β, putative pyruvate kinase, putative sugar oxidoreductase, hexokinase 2, glycerol 3-phosphate dehydrogenase, actin, glutamine synthetase, aldo/keto reductase, thioredoxin reductase, peroxiredoxin.  **Up-regulated (59/110):** Probable ATP binding protein, pyruvate decarboxylase, HSP70, putative pyruvate decarboxylase, enolase, putative phosphoglycerate dehydrogenase, 6- phosphogluconate dehydrogenase, glyceraldehyde-3-phosphate dehydrogenase, alcohol dehydrogenase, 3-isopropylmalate dehydrogenase, fructose-bisphosphate aldolase , enolase, 40S ribosomal protein S5. |
| 25 | Rich medium, mid-exponential phase  was subjected to oxidative stress H_2_O_2_/DIGE | 2D gel  strip 3-10  and 4-7  MALDI MS | *S. pombe* | -Total proteins observed: 1040; analyzed: 158 | -57% of differentially regulated proteins corresponded to four functional groups; glycolysis (19%), amino acid metabolism (16%), molecular chaperones (11%) and protein synthesis (11%).  -11% were of unknown function.  -The remaining 32% represented 15 different functional categories.  -Proteins involved in glycolysis represented the largest functional group, with 50 isoform entries (14 gene products). Enzymes in the upper half of the pathway were up-regulated, while those in the lower half were down-regulated.  -32 oxidoreductases were identified. | **Down-regulated :** Alcohol dehydrogenase 1, enolase, fructose 1,6-bisphosphate aldolase, hexokinase 1,phosphoglucomutase precursor, pyruvate dehydrogenase, phosphoglycerate kinase, 2-hydroxy acid dehydrogenase, acetolactate synthase, hydroxymethylglutaryl-CoA synthase, NADP-specific glutamate dehydrogenase, ornithine aminotransferase, Ade1, min4, phosphoribosyl amine glycine, adenylosuccinate synthetase, hsc1, sks2, HSP 70 family, ssc1, ssp1, F1-ATP synthase, ribose 5-phosphate isomerase.  **Up-regulated:** Glyceraldehyde-3-phosphate dehydrogenase 1, Pfk1, 6-phosphofructokinase, pyruvate kinase, aldo/keto reductase , dihydroxyacetone kinase 1, dak1, glucose-6-phosphate 1-dehydrogenase, zwf1, glutamine synthetase, glutamate-ammonia ligase, glycerol-3-phosphate dehydrogenase, malate dehydrogenase, phosphoglucomutase precursor, phosphoglycerate dehydrogenase, transaldolase, transketolase , catalase, glutathione *S*-transferase 2, thioredoxin peroxidase, vacuolar ATP synthase catalytic subunit α, succinate semialdehyde dehydrogenase (predicted), zinc-binding dehydrogenase, T-complex protein 1, alpha subunit. |
| *** | YPD medium, exponential growth  phase (5, 15,  30, or 60 min after addition of H_2_O_2_ or diamide/ silver nitrate | 2D gel  strip 3-10  MALDI-TOF MS | *C. albicans* | -Total proteins observed: ND; analyzed: 52 | Proteins were involved in a broad range of functions, including ROS detoxification, C-compound and carbohydrate metabolism, amino acid metabolism, oxidoreduction, chaperones, nucleotide metabolism, protein synthesis and other. | **Down-regulated :**  -Amino acid metabolism (4): Pentafunctional enzyme, putative sulfite reductase,histidine biosynthesis trifunctional protein, cysteine synthase.  -C-compound and carbohydrate metabolism (9): Aconitate hydratase, citrate synthase, aldohexose-specific glucokinase, hexokinase II, pyruvate dehydrogenase, 6-phosphofructokinase, pyruvate carboxylase 2, UTP-glucose-1-phosphate uridylyltransferase.  -Nucleotide metabolism (2): Adenylosuccinate synthetase, 5-phosphoribosylformyl glycinamidine synthetase.  -Protein synthesis (13): Protein of the 40S ribosomal subunit, aspartyl-tRNA synthetase, cysteinyl-tRNA synthetase, methionyl-tRNA synthetase.  -Other (12): Acetyl-coenzyme-A hydrolase, F1F0-ATPase complex, F1 α subunit, stress protein, probable quinone oxidoreductase, nuclear pore protein, ABC transporter, oxidoreductase, DnaK-type molecular chaperone.  **Up-regulated:**  -ROS detoxification (8): Cadmium-induced protein, catalase A, glutathione reductase, glutathione peroxidase, TSA/alkyl hydroperoxide peroxidase C (AhPC) family, putative alkyl hydroperoxide reductase, GST, thioredoxin reductase.  -Oxidoreductases (8): NADPH dehydrogenase, putative reductase, potential zinc-binding dehydrogenase, alcohol dehydrogenase, putative NADH-dependent flavin oxidoreductase.  -Chaperones (5): Heat shock protein, HSP70 family.  -Other (11): D-Arabinose dehydrogenase, argininosuccinate synthetase, GTP-binding protein, putative esterase, mitochondrial respiratory function protein, serine hydroxymethyltransferase precursor, mitocondrial stress-induced protein. |
| 31 | YEPD medium, exponentially growing. To induce apoptosis, H_2_O_2_ was added and cells were collected after 3 h/ silver staining | 2D gel  strip 3-10  MALDI-TOF MS and ESI-IT MS | *S. cerevisiae* | -Total proteins observed: 2000; analyzed: 31 | The differentially expressed proteins were sorted into the following functional groups: fermentative pathway (4), glycolysis (14), amino acid and pyrimidine metabolism (4), heat shock (3), and others (6). | Exposure of yeast cells to non-physiological doses of peroxides decreases the expression (or increases the degradation) of enzymes involved in protecting against oxidative stress leading to a reduced level of antioxidant defense and making the cells more prone to apoptosis.  **Down-regulated:** Enolase 2, triosephosphate isomerase, glyceraldehyde-3-phosphate dehydrogenase 2-3, homoserine dehydrogenase, alcohol dehydrogenase I, peroxiredoxin.  **Up-regulated:** Transketolase 1, pyruvate decarboxylase 1, homocitrate synthase, heat shock protein 75, pyruvate kinase 1, enolase 2, fructose-bisphosphate aldolase, 3-isopropylmalate dehydratase, glyceraldehyde-3-phosphate dehydrogenase 3, mannose-1-phosphate guanyltransferase, cytocrome *c* oxidase, heat shock protein SSA2. |
| 24 | YPD medium, exponential and stationary growth phase/ silver or colloidal Coomassie stained | 2D gel  strip 3-10  MALDI-TOF MS | *C. albicans* | -Total proteins observed: ND; analyzed: 360 | -50 most abundant proteins on colloidal Coomassie stained 2-D gels in exponential and stationary growth. | The stationary-phase signature revealed a complex reprogramming of metabolic networks. Up-regulation of the glyoxylate cycle, gluconeogenesis and glutamate degradation signal a switch to the utilization of alternative carbon sources instead of the exhausted glucose. The induction of proteins involved in defending against oxidative and heat stress indicates a change in the redox balance and in the concentrations of reactive oxygen species.  **Exponential phase:**  -Amino acid metabolism (4/4): Ornithine aminotransferase, ketol-acid reducto-isomerase, threonine synthase.  -C-compound and carbohydrate metabolism (9/29): Aconitate hydratase, alcohol dehydrogenase, acetyl-coenzyme-A synthetase, fructose-bisphosphate aldolase, 6-phosphogluconate dehydrogenase, NAD-isocitrate dehydrogenase, pyruvate dehydrogenase, phosphomannomutase, transaldolase.  -Cell rescue, defense and virulence: (4/11): Likely thioredoxin peroxidase, ubiquitin-activating enzyme.  -Energy (3/6): NADH-cytochrome-b5 reductase, dihydrolipoamide dehydrogenase.  -Protein fate (6/6): Aminopeptidase yscII, microsomal ATPase, HSP70 family.  -Protein synthesis (5/6): Protein of the 40S ribosomal subunit, ribosomal protein L10.  -Other (8/10): Actin, adenosine kinase, likely metallopeptidase, S-adenosyl-L-homocysteine hydrolase.  **Stationary phase:**  -C-compound and carbohydrate metabolism (20): Alcohol dehydrogenase, alcohol dehydrogenase I, pyruvate kinase, citrate synthase, enolase I, fructose-bisphosphate aldolase, glyceraldehyde-3-phosphate dehydrogenase, aldohexose-specific glucokinase, phosphoglycerate mutase, isocitrate lyase, mitochondrial malate dehydrogenase precursor, pyruvate decarboxylase, glucose-6-phosphate isomerase, phosphoglycerate kinase, transketolase 1.  -Cell rescue, defense, and virulence: (7/11): Stress protein, HSP60, superoxide dismutase, HSP70.  -Energy (3/6): NAD-aldehyde dehydrogenase, ATPase F1 α subunit.  -Other (2/10): Acetyl-coenzyme-A hydrolase, unknown function. |
| This study | MM glucose 2%, lag, exponential and stationary phase/ Coomassie stained | 2D gel  strip 3-10  MALDI-TOF MS | *X. dendrorhous* | -Total proteins observed: 600; analyzed:131 | -Genetic information processing (25%)  -Environmental information processing (5%)  -Cellular processes (13%)  -Unknown 8%  -Metabolism proteins (49 %), consisting of the following subgroups:  -carbohydrate (13%), redox (8%), amino acid (11%), energy metabolism (2%), lipid (5%), carotenoid biosynthesis (6%), other (5%). | -Prior to carotenogenesis, enzymes related to acetyl-CoA synthesis were present at higher abundances. Later, redox- and stress- related proteins were up-regulated during the induction of carotenogenesis. For the carotenoid biosynthetic enzymes mevalonate kinase and phytoene/squalene synthase, we observed higher abundances during the induction and/or accumulation of carotenoids.  -The most abundant proteins found in this study were involved in metabolic pathways and were present in multiple spots.  -The expression patterns of carbohydrate and lipid metabolism proteins demonstrated a tight regulation of substrates for cellular metabolism.  -Redox proteins were up-regulated to contribute to late stage astaxanthin synthesis reactions. |

Ref., reference.

* Hwang KH, Carapito C, Böhmer S, Leize E, Van Dorsselaer A, Bernhardt R: **Proteome analysis of *Schizosaccharomyces pombe* by two-dimensional gel electrophoresis and mass spectrometry.** Proteomics 2006, **6:**4115-29.

** Weeks ME, Sinclair J, Jacob RJ, Saxton MJ, Kirby S, Jones J, Waterfield MD, Cramer R, Timms JF: **Stress-induced changes in the *Schizosaccharomyces pombe* proteome using two-dimensional difference gel electrophoresis, mass spectrometry and a novel integrated robotics platform.** Proteomics 2005, **5:**1669-85.

***Kusch H, Engelmann S, Albrecht D, Morschhäuser J, Hecker M: **Proteomic analysis of the oxidative stress response in *Candida albicans*.** Proteomics 2007, **7:**686-97.
